# Large-effect loci mediate rapid adaptation of salmon body size after river regulation

**DOI:** 10.1073/pnas.2207634119

**Published:** 2022-10-24

**Authors:** Arne J. Jensen, Ingerid J. Hagen, Yann Czorlich, Geir H. Bolstad, Gunnbjørn Bremset, Bengt Finstad, Kjetil Hindar, Øystein Skaala, Sten Karlsson

**Affiliations:** ^a^Norwegian Institute for Nature Research, NO-7485 Trondheim, Norway;; ^b^Department of Biology, Norwegian University of Science and Technology, NO-7491 Trondheim, Norway;; ^c^Institute of Marine Research, NO-5817 Bergen, Norway

**Keywords:** evolution, adaptation, *vgll3*, *six6*, large-effect loci

## Abstract

Understanding the potential of natural populations to adapt to altered environments is becoming increasingly relevant in evolutionary research. Currently, our understanding of adaptation to human alteration of the environment is hampered by lack of knowledge on the genetic basis of traits, lack of time series, and little or no information on changes in optimal trait values. Here, we used time series data spanning nearly a century to investigate how the body mass of Atlantic salmon (*Salmo salar*) adapts to river regulation. We found that the change in body mass followed the change in waterflow, both decreasing to ∼1/3 of their original values. Allele frequency changes at two loci in the regions of *vgll3* and *six6* predicted more than 80% of the observed body mass reduction. Modeling the adaptive dynamics revealed that the population mean lagged behind its optimum before catching up approximately six salmon generations after the initial waterflow reduction. Our results demonstrate rapid adaptation mediated by large-effect loci and provide insight into the temporal dynamics of evolutionary rescue following human disturbance.

## Significance

Many ecosystems are altered by human activity. To survive, affected populations must respond to these changes by means of adaptive plasticity or adaptive trait evolution. Allele frequency changes at functional loci provide evidence of evolutionary response, but such documentation is scarce in wild populations. Using concordant temporal genetic and phenotypic data from a wild population of Atlantic salmon impacted by hydropower development, we present one of the largest evolutionary changes reported on the decadal timescale. Modeling the dynamics revealed a rapid adaptive response to the reduced waterflow. Our results provide clear evidence of human-induced evolution mediated by large-effect loci in a wild population.

While extinction of populations affected by human activity is common (1), some populations manage to adapt and survive. Detailed studies of these successful evolvers are warranted to identify the potential for evolutionary rescue (2). Many studies document phenotypic change following human activities (3). However, with the exception of studies on introductions and pollution, few of these have isolated the genetic response from plastic responses (3), and even fewer have associated phenotypic responses with molecular genetic responses (4–7). By combining long time series on phenotypic and environmental change with knowledge on genetic architecture, it is possible to get an understanding of the dynamics of contemporary evolution by modeling how the optimal phenotype changes and how this causes molecular and phenotypic responses. Because of sparse knowledge about the underlying genetic architecture of focal traits and the need of long time series, such studies are challenging in wild populations.

The Atlantic salmon (*Salmo salar* L.) provides a rare case where we have knowledge on the genetic basis of an important life history trait: number of years spent at sea before maturing, or “sea age.” The genetic architecture of sea age involves two major effect loci, one in the genomic region *vgll3* on chromosome 25 and the other in the genomic region *six6* on chromosome 9, both explaining a substantial fraction of the observed trait variation (8–10). Longer time spent at sea correlates with increased body mass (11) and subsequent higher reproductive success (12), but at the cost of increased risk of dying before reproduction (11). A positive relationship between waterflow and the average adult body size of Atlantic salmon inhabiting different rivers (13, 14) suggests that waterflow is a selective agent affecting the optimal trade-off between marine survival and reproduction. River size and waterflow are important habitat characteristics frequently altered by human activities (15, 16), causing changes to which populations of Atlantic salmon and other freshwater species must adapt or go extinct.

River Eira (62°41′N, 8°7′E) in western Norway once harbored salmon that averaged 12 kg and were among the largest in the world (17). During the last century, River Eira was one of many rivers that were developed for hydroelectric purposes. Over three consecutive hydropower developments in 1953, 1962, and 1975, water from River Eira’s catchment area was transferred to hydropower stations in neighboring watercourses. As a result, the waterflow in River Eira was reduced to 60%, 50%, and, finally, 40% of its original flow, presumably imposing a different selection pressure on the salmon population in the river. Indeed, the average salmon in River Eira is now ∼4 kg, a third of its historical value (18). We hypothesized that this reduction in size is a result of evolutionary response to reduced waterflow, mediated by changes in the allele frequencies at two functional loci in the regions of *vgll3* and *six6*. Additionally, we discuss three alternative explanations that these changes were induced by stocking, changes in the marine environment, or size-selective harvest. We used date and mass of 8,324 adult Atlantic salmon caught by anglers in River Eira during the periods 1925–1926 and 1940–2016. Scale samples were available for a subset of these salmon, and we analyzed DNA extracted from 346 scale samples. Equivalent data from salmon caught during an overlapping period in another river, also known for its large salmon but without any reduction in waterflow, allow for inferences on possible effects of the marine environment, which is common for the two populations. We quantify the adaptive dynamics of the River Eira salmon by jointly modeling changes in genotype, phenotype, and phenotypic optimum over a period of 77 y (∼13 salmon generations).

## Results

### Phenotypic Change.

**Fig. 1.**
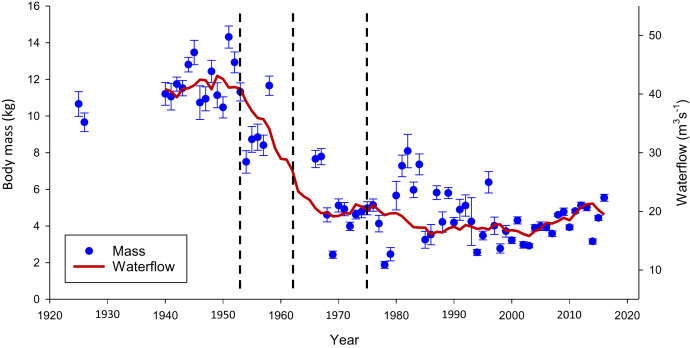
Changes in waterflow and body mass of Atlantic salmon in River Eira. The 10-y running mean of annual waterflow in River Eira (red line) from 1940 to 2016 and the mean body mass (kilograms) of individual Atlantic salmon caught in River Eira (blue filled circles with bars denoting ±1 SE) from 1925 to 2016. Dashed vertical lines indicate the timing of the three different hydropower developments.

Prior to the hydropower developments in River Eira, the mean body mass of Atlantic salmon in the reported catches was 11.68 ± 0.15 kg, ranging between 1 and 28 kg (Fig. 1 and *SI Appendix*, Table S1). Already, in the first run season (1954) after the initial waterflow reduction, the average body mass was 7.5 ± 1.22 kg, which constitutes an instant reduction to 64% of the previous 14 run seasons. During 1940–1953, 53 individuals (5.9%) of 20 kg or more were reported in the catches, while only two individuals (<0.1%) of 20 kg or more have been reported since 1953. In the interim between and after the waterflow reductions, the mean body mass of salmon in reported catches was reduced to 74%, 41%, and 38% of the original mass (Fig. 1 and *SI Appendix*, Table S1). These observations translate into a tight relationship between waterflow and body mass (*r*^2^ = 0.60; *SI Appendix*, Fig. S1). The decrease in body mass from before 1953 to after 1975 corresponds to a change of −45,454 darwins, −0.49 haldanes, or −1.0 natural log units (“the darwin numerator”). Compared with a recent metaanalysis on contemporary evolution (3), this change is among the 5% largest changes observed. The decrease in body mass was due to decreases in sea age as well as size within sea age. For mean body mass between 1925–1926 and between 1987–2016, the periods for which data on both sea age and body mass were available, 46% could be attributed to reduced sea age, and 54% could be attributed to reduced body mass within sea age (*SI Appendix*, Table S2).

### Allele Frequency Changes.

**Fig. 2.**
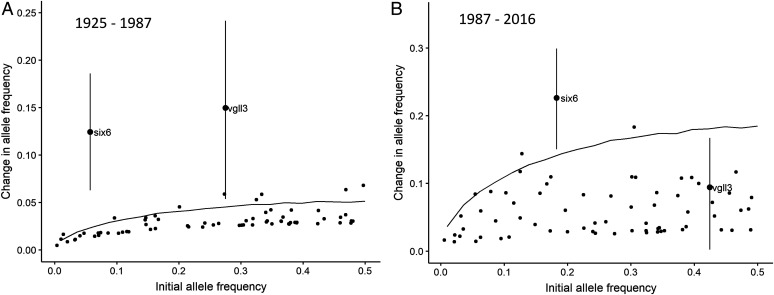
Absolute allele frequency changes for the genes *vgll3* and *six6* over time in Atlantic salmon from River Eira in comparison with putatively neutral markers. Each dot represents an SNP marker. The genes *vgll3* and *six6* are indicated. The remaining 67 neutral markers represent genetic drift. The solid line represents the absolute amount of change expected under drift at the 95th quantiles. Vertical bars represent 95% credible intervals. (*A*) Changes in allele frequency for the genes *vgll3* and *six6* comparing samples collected during the years 1925–1926 and 1987. (*B*) Changes in allele frequency for the genes *vgll3* and *six6* comparing samples collected in 1987 and 2016.

The respective “early” maturation alleles of *vgll3* and *six6* are associated with a lower sea age and body mass at sexual maturity, while the “late” alleles are associated with a higher sea age and body mass at sexual maturity (9). To test for changes in the frequency of the “early” and “late” alleles, we used samples collected in River Eira at three different periods (1925–1926, 1987, and 2016). Changes at an additional 67 neutral single-nucleotide polymorphism (SNP) markers (19) represented genetic drift. There were large allele frequency changes suggesting strong selection on the genes *vgll3* and *six6*. The *vgll3* early maturation allele (*E_vgll3_*) frequency increased from 0.28 to 0.43 (i.e., a change of 0.15, CI_95%_ = 0.05 to 0.24), while the *six6* early maturation allele (*E_six6_*) increased from 0.06 to 0.18 (i.e., a change of 0.12, CI_95%_ = 0.06 to 0.19) between samples collected prior to the hydropower developments (1925–1926) and samples collected 34 y after the first waterflow reduction (1987) (Fig. 2 and *SI Appendix*, Fig. S2). Both *vgll3* and *six6* allele frequency changes were unlikely to be due to drift (mean probabilities of 0.5% and 0.1%, respectively). A further allele frequency change observed from 1987 to 2016 in *vgll3* had a higher probability to be due to drift alone (probability of 34%, change of 0.09, CI_95%_ = 0.01 to 0.18), while the consistent and statistically significant allele frequency change from 1987 to 2016 in the *six6*-linked marker indicated continued selection on this marker (Fig. 2; drift probability of 0.8%, change of 0.22, CI_95%_ = 0.15 to 0.30). The lack of evidence for adaptive evolution in *vgll3* in the latter period (1987–2016), during which neither waterflow nor body mass changed drastically, is not surprising. The continued change of *six6* may reflect ongoing selection on other associated traits, such as run timing (20, 21). Analyses of Atlantic salmon caught in the nearby River Stryn, also harboring large-sized salmon, revealed no change in mass and no significant temporal changes in allele frequencies of *vgll3* and *six6* (*SI Appendix*, Figs. S3 and S4), thus supporting the hypothesis that the observed evolutionary response in River Eira was caused by reduced waterflow as opposed to changes in the marine environment.

### Functional Effect of *vgll3* and *six6*.

We estimated the within-year sex-specific effect of *vgll3* and *six6* on individual log mass using linear regression. The *vgll3* marker was strongly associated with mass of Atlantic salmon: Males and females being homozygous for the late maturation allele on *vgll3* (*LL_vgll3_*) were, respectively, 3.77 (CI_95%_ = 2.78 to 5.12) and 2.37 (CI_95%_ = 1.94 to 2.88) times larger than individuals being homozygous for the early maturation allele (*EE_vgll3_*) genotype, with reversed dominance between the sexes (*SI Appendix*, Fig. S5 and Table S3). Also, the *six6* marker showed strong association with mass: Males and females carrying the *LL_six6_* genotype were, respectively, 1.76 (CI_95%_ = 1.13 to 2.77) and 1.94 (CI_95%_ = 1.55 to 2.42) times larger than individuals with *EE_six6_* genotype (*SI Appendix*, Fig. S5 and Table S3). The allele frequency change in *vgll3* and *six6* contributed substantially to the observed decrease in body mass in River Eira. These genetic changes predicted 84% and 81% of the observed change in mass from 1925 to 2016 in females and males, respectively. The corresponding proportions of body mass reduction predicted by genetic change from 1925 to 1987 were 46% in females and 59% in males.

### Potential Effects of Stocking.

To determine whether the stocking program, which started its releases in 1959, contributed to the genetic changes in the Eira salmon population, we first compared the differences in *vgll3* and *six6* allele frequencies between naturally produced individuals and hatchery-produced individuals from the 1987 and 2016 run years. The allele frequency difference between hatchery-produced and wild salmon varied idiosyncratically between years (*SI Appendix*, Fig. S6). Across years, the hatchery-produced fish had, on average, lower *E* allele frequency compared with naturally produced fish, but the difference was not statistically significant (log-odds difference ± SE: −0.07 ± 0.31, *P* = 0.86 and −0.30 ± 0.14, *P* = 0.26 for *vgll3* and *six6*, respectively). Hatchery-produced and naturally produced individuals caught during 1987–2016 were also of the same size (mean mass ± SE of 4.29 ± 0.05 and 4.31 ± 0.06 kg, respectively). Second, allele frequencies in the first hatchery-produced individuals caught at sea in 1961–1967 (stocked as smolts 1959–1964) were similar to the wild individuals caught in 1925–1926 (difference of 0.06, CI_95%_ = −0.04 to 0.15 and −0.05, CI_95%_ = −0.11 to 0.01 for *E_vgll3_* and *E_six6_*, respectively; *SI Appendix*, Fig. S6). Third, a comparison of broodfish used for stocking with all fish caught by anglers revealed a preference for broodfish with a larger size than the population average (*SI Appendix*, Fig. S7). Together, these results show that stocking cannot explain the large increase in *E* allele frequency and concordant body mass reduction of the River Eira salmon.

### Modeling the Adaptive Dynamics.

Using quantitative genetic theory in combination with statistical state-space modeling, we estimated the joint temporal dynamics of mean mass, its optimal value, and the allele frequency changes at the two major effect loci. The model assumed quadratic stabilizing selection around a changing optimum, where the optimum dynamics was given by a log-linear function of the average waterflow during the salmon run (June to September). The evolutionary potential was determined by the genetic variance of mass, calculated as a function of the allele frequencies of *vgll3* and *six6* in addition to a component of unobserved small-effect genes. We fitted the model to the data using maximum likelihood. Despite its highly simplified relationship between phenotype and fitness, the model explained 65% of the observed yearly variation in mean mass. Of the nonexplained fraction, 2% was due to measurement error, while the remainder (33%) could be attributed to nongenetic changes (i.e., plasticity), genetic changes due to drift, and/or selection not included in the model.

**Fig. 3.**
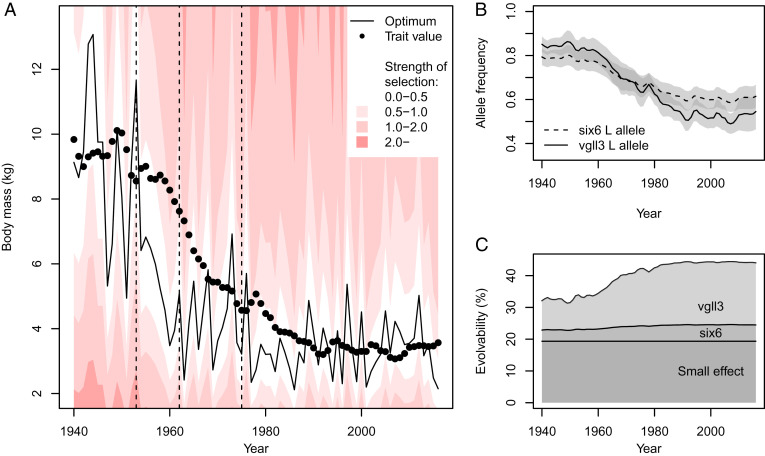
Adaptive dynamics and evolution of salmon body mass in River Eira. (*A*) The changing adaptive landscape and evolution of mean log body mass (genetic value before selection). Declining fitness from the fluctuating optimum and increasing steepness of the selection gradient is indicated by shades of red. Strength of selection is in units of inverse natural log mass and is therefore comparable to mean standardized selection gradients [i.e., in units of selection on fitness itself (22)]. Vertical dashed lines show years of hydropower development. (*B*) Evolutionary dynamics of the allele frequencies at the functional loci. The *L* allele is associated with large body size. The 95% CIs are shown in gray. (*C*) Temporal dynamics of the evolvability, with the contribution of unobserved small-effect genes, *six6*, and *vgll3* stacked on top of each other. Evolvability, the percent increase of the trait mean under unit selection (*β* = 1) (23), is given by 100 × (*e*^Va^ -1), where V_A_ is the additive genetic variance on the natural log scale. See *SI Appendix*, Table S4 for parameter estimates and *SI Appendix*, Table S5 for model output.

The model estimated a proportional relationship between the optimal trait value and waterflow (optimum ∝ waterflow^1.00±0.11^) and shows that the average trait value lagged behind the fluctuating optimum before catching up in the late 1980s (Fig. 3*A*). Assuming a generation time of 6 y in Eira, the successful adaptation to a new optimum took about six salmon generations from the initial waterflow reduction. According to our model, selection for smaller size was strong in the period after the first waterflow reduction, but with large fluctuations in magnitude (Fig. 3*A*). The change in average trait size was mediated by the change in allele frequencies of *vgll3* and *six6* (Fig. 3*B*), where the genetic variation at these loci contributed substantially to the high potential for evolution, or evolvability (Fig. 3*C*). The adaptation was associated with a considerable genetic load (fitness reduction) in the period of hydropower development (*SI Appendix*, Fig. S8).

An important assumption of our model is the constant age structure of the returning adults. Modeling the evolution of the age structure was not feasible, due to lack of data. The age structure we used was based on all age data, in which most individuals were from 1987 onwards and had a considerably younger age (∼2 y) compared to the individuals caught in 1925–1926 (*SI Appendix*, Table S1). If we shift the age structure upwards by 2 y in the model, the adaptive dynamics changes so that the mean trait value catches up with its optimum in the mid-1990s (*SI Appendix*, Fig. S9). Hence, even with this long generation time, the salmon adapts quickly to the altered waterflow. *SI Appendix*, Figs. S10–S13 present more analyses on assumptions of the model.

While our model relies on several assumptions and can only crudely quantify the evolutionary process, a major benefit of our modeling approach is that it gives a quantitative assessment of the results in a theoretical context. Given the estimated evolvability and strength of selection, the model shows that adaptation to a new optimum is possible within the time series. Such quantification is often neglected but highly important for understanding the evolutionary process (24).

## Discussion

The observed decline in mass over the two decades of hydropower development is among the largest evolutionary changes reported on the decadal timescale in metaanalyses (3, 25). An ability to evolve is a necessity for evolutionary rescue and population persistence during environmental changes (26). However, as lineage extinction is the most common outcome for taxa (27), it is evident that evolutionary responses often do not keep up with environmental change. Several factors have likely facilitated the rapid adaptation in the River Eira salmon population. First, trait evolvability was very high and largely caused by two large-effect loci (Fig. 3*C*) where genetic variation may have been maintained at a high level by fluctuating (28) or balancing selection (9). Second, selection was toward an intermediate allele frequency at the large-effect loci (Fig. 3*B*), thereby increasing evolvability during the evolutionary response (Fig. 3*C*). If selection had favored more extreme allele frequencies, evolvability would have decreased, and the rate of adaptation would have slowed down. Third, selection for smaller size in River Eira indirectly selected for younger sea age and therefore increased survival at sea. Hence, there seems to have been concordant selection on reproduction and survival during the period of adaptation, promoting faster evolutionary response by alleviating potential pleiotropic constraints (29). A likely scenario is that large fish did not enter the breeding ground and therefore suffered severely reduced reproduction.

It is unlikely that other factors than the waterflow reduction were main drivers of the observed phenotypic and genetic changes. First, even though marine selection influences the body mass of mature Atlantic salmon (5, 30), it cannot explain why River Eira salmon experienced a large reduction in body mass during 1954–1974 (Fig. 1), while body mass remained stable in River Stryn, another population with large salmon using the same marine environment. A significant regime shift in the marine ecosystem from 2004 caused a substantial reduction in marine growth of many salmon populations in western Norway (30), and, while this occurred within our study period, it does not overlap with the period of the largest body mass reduction in River Eira. Second, release of hatchery-produced fish did not contribute to the initial phenotypic changes observed in River Eira, as the initial releases began in 1959, at which point the mean body mass was already reduced to 74% of mean mass prior to the waterflow reductions. It is also unlikely that stocking contributed to the observed genetic changes. Stocked individuals recaptured during 1961–1967 did not show increased frequencies of the early alleles of *vgll3* and *six6,* and stocked individuals from 1987 and 2016 did not differ in *vgll3* and *six6* allele frequencies compared with naturally produced fish. While affecting population genetic parameters, such as reduced effective population size following a Ryman–Laikre effect (31, 32) and subsequent increased genetic drift (Fig. 2) after 2006, when the fraction stocked fish has been very high (31), the stocking cannot explain the large reduction in mean body mass of salmon during the hydropower development (1953–1975) in River Eira. Third, it is unlikely that harvest (angling in River Eira and bag nets at sea) induced the observed response: The fishing pressure was strong prior to the first waterflow reduction in the Eira (*SI Appendix*, Fig. S14), and similar fishing pressure in other rivers did not cause a reduction in body size in the local populations (e.g., River Stryn; *SI Appendix*, Fig. S14 and S3). Finally, our adaptive dynamics model explained well the observed body mass, suggesting that the phenotypic changes in River Eira were caused by the altered waterflow.

Our study provides unequivocal evidence of unintentional human-induced evolution in a natural population, in which the species managed to adapt to the altered environment. River Eira once harbored some of the largest salmon in the world but has now evolved into an ordinary salmon population. This successful adaptation comes at a cost of reduced life-history diversity, and, potentially, reduced population stability and resilience to further environmental change (33).

## Materials and Methods

### The Study System.

The focus of this study is an Atlantic salmon population inhabiting River Eira, situated in a mountainous area of western Norway at 62°41′N, 8°7′E. Its waterflow, which had a historical annual average of 41 m^3^⋅s^−1^, was reduced to 17 m^3^⋅s^−1^ by three separate hydropower developments in 1953, 1962, and 1975, each removing water from the watershed by transferring parts to neighboring watersheds (34) (Fig. 1). While the waterflow peaks in summer and is at a low during winter, the waterflow reduction is proportionally similar throughout the year (*SI Appendix*, Fig. S15).

To compensate for reduced juvenile production, River Eira has, since 1967, been stocked annually with ∼50,000 2-y-old hatchery-produced Atlantic salmon smolts of local origin (35). A smaller number of smolts were released in the years 1959–1966, these numbers being 1,065, 8,520, 4,181, 10,778, 11,194, 6,003, 5,507, and 39,180 smolts, respectively. A proportion of these were tagged with individually numbered Carlin tags (36), and scale samples were taken from recaptured fish for further analyses. The fraction of stocked fish in catches of adult salmon has increased with time but was not recorded prior to 1987. In the years 1987 and 2016, 12% and 51% of the individuals were of stocked origin, while the remainder were naturally produced. New broodfish has been caught from the evolving river population for each new generation of hatchery-produced fish.

To test whether the observed evolutionary change in the River Eira salmon population was a response to altered selection pressure at sea, we included samples from another river famous for its large salmon, where the waterflow has not been changed, the River Stryn. River Stryn is located on the west coast of Norway at 61°54’N, 6°42’E, ∼115 km southwest of River Eira, and has an annual waterflow of 32 m^3^⋅s^−1^. The Atlantic salmon population in River Stryn migrate to and forage in the same marine areas as do the River Eira population (37). Atlantic salmon home to their natal rivers to spawn, and the salmon in these two rivers constitute different populations, with a pairwise genetic distance at neutral loci of 0.029 (Weir and Cockerham’s F_ST_) (38), which has remained stable over the study period.

### Catch Information, Scale Samples, and Molecular Analysis.

Anglers in River Eira were requested to record date of catch and size (mass and length) of each adult salmon captured by rod during the sport fishing season. From 1925 until 2016, all sizes and both sexes were fished and culled indiscriminately. Nearly all (99%) of the catches in our data were taken between 1 June and 8 September. We used information on catch year and individual mass of 8,324 adult Atlantic salmon caught by anglers in the periods 1925–1926 and 1940–2016 (except 1958–1965, from which catch information is missing). During 1925–1926, and annually since 1987, anglers also collected fish scales from their catch. Experienced scale readers recorded number of years spent in freshwater (smolt age) and number of years at sea (sea age) and identified hatchery-produced individuals according to growth patterns (annuli) in the scale (39). From 2001 onward, the adipose fin was removed from all hatchery-produced smolt; hence, after 2002, anglers were also requested to provide information about presence/absence of the adipose fin. Because naturally produced and hatchery-released fish could not be differentiated prior to 1987, we included both naturally produced and hatchery-released fish when constructing the time series of average body mass. For the years 2005–2016, we also had the mass of fish selected and used as broodfish to produce juveniles for hatchery releases. Data on broodfish were only used to compare body mass with fish caught by anglers and were not included on the time series of average body mass.

We extracted DNA from a subset of the scale samples belonging to three periods: 1925–1926 (*n* = 77), 1987 (*n* = 120), and 2016 (*n* = 149), for genotyping and genetic sex determination. To investigate the allele frequencies of the *vgll3* and *six6* markers in hatchery-produced fish at a time when the body mass of salmon in River Eira was declining, we also extracted DNA from scales of 79 Carlin-tagged individuals that were recaptured during 1961–1967 as sexually mature adults close to shore along the Norwegian coast while on their home migration. The individuals were mainly caught by bag nets, which efficiently catch all sea ages of the River Eira salmon. For River Stryn, we found equivalent genetic and phenotypic data from the periods 1955–1957 (*n* = 69), 1983 (*n* = 78), and 1990 (*n* = 67). DNA was extracted from the scales using DNEASY tissue kit (QIAGEN) and genotyped on the EP1 96.96 Dynamic array IFCs platform (Fluidigm) at 68 neutral nuclear SNPs (*SI Appendix*, Table S6), as well as two SNPs in linkage with the functionally important genes *vgll3* and *six6*, respectively (*SI Appendix*, Table S6). The sex was determined using the sdY gene (40) amplified in one multiplex together with genetic markers for differentiating between Atlantic salmon and brown trout (*Salmo trutta*) (41). The samples collected in 2016 were initially filtered to remove individuals with possible ancestry in farmed escapees (42) (31 individuals removed). In the early period of Atlantic salmon aquaculture (prior to 1990), farmed genetic introgression was insignificant, and samples from these earlier years are being used as references for wild Atlantic salmon (43). Introgression is therefore not expected to affect the samples from 1990, or earlier years. Individuals with genotyping success below 0.7 were removed from further analysis.

### Allele Frequency Changes.

To determine whether temporal changes in *vgll3* and *six6* allele frequency in the Eira and Stryn populations could be explained by genetic drift, we used an approximation of the Wright–Fisher model (44, 45). The number of alleles at each putatively neutral locus *i* in time *t* followed a binomial distribution to account for sampling error

$$A_{i,t\;}∼\mathrm{Bin}(x_{i,t},\;T_{i}),$$

where *T_i_* is the total number of alleles at the locus *i*, and *x*_(_*_i,t_*_)_ is the allele frequency at period *t*. Initial allele frequency in the model, *x_i,t_*, followed a beta distribution with shape parameters equal to one. Otherwise, the allele frequency *x_i,t_* was derived from the previous period, *x_i,t-_*_1_, using a normal distribution (46),

[1] $$x_{i,t}|x_{i,\;t-1}∼N\left(x_{i,\;t-1},\;x_{i,\;t-1}\left(1-x_{i,\;t-1}\right)\frac{g_{t}}{2N_{t}}\right),$$

where $g_{t}$ is the number of generations between the periods *t* and *t* − 1, and $N_{t}$ is the effective population size. We estimated the ratio ζt=gt2Nt in a Bayesian model with a prior uniform distribution ranging from 0.0001 to 1 (e.g., ref. 47) by using the Just Another Gibbs Sampler software (JAGS) and by including the “dinterval” function to ensure that $x_{\left(i,t\right)}|x_{\left(i,\;t-1\right)}$ ranged between zero and one (48). We ran two Monte Carlo Markov chains (MCMCs) in R (R core team) for 770,000 iterations, including a burn-in length of 370,000 iterations, and kept 1 out of 40 iterations. To assess convergence, we used the Gelman and Rubin’s convergence diagnostic (49) and a potential scale reduction factor threshold of 1.1. In the same model, we derived the *six6* and *vgll3* allele frequencies in Rivers Eira and Stryn at each period using a beta-binomial distribution. Shape parameters from the beta distribution were set to one as a prior. We calculated the difference in allele frequency between consecutive periods at each iteration and compared with the variation expected under drift from Eq. **1**, using the estimate of $\zeta_{t}$ (1925/1926 to 1987: 0.0001, 0.0019, 0.0058 and 1987–2016: 0.0185, 0.0313, 0.0478 for lower 95%, median, and upper 95%, respectively). Because fixed putative neutral SNP markers were excluded, we used 67 such markers in River Eira analyses and 66 in River Stryn analyses.

### Functional Effect of *vgll3* and *six6*.

To study the within-year phenotypic effect of the two loci, we used the natural genetic effect model (50). The model had log mass (ln kilograms) as a response variable and included both sex-specific additive (*a*) and dominance (*d*) effects. Due to low sample size of one genotype (*EE_six6_*), we did not estimate *d* for *six6*. To control for temporal effects, we centered the explanatory variables on their within-year means for each sex. In addition, we included the catch period as a fixed factor in interaction with sex. To obtain 95% CIs, we used Monte Carlo simulation by drawing 50,000 samples from a multivariate normal distribution, specified by point estimates and their error variance matrix. Fish with hatchery origin was excluded from this analysis.

### Potential Effects of Stocking.

To estimate *vgll3* and *six6* allele frequencies and their 95% credible intervals in wild and hatchery-produced fish per sampling period, we used beta-binomial models in JAGS with priors for the beta shape parameters set to one. We ran two MCMC chains for 50,000 iterations, including a burn-in length of 50,000 iterations, and kept 1 out of 5 iterations. The posterior distribution of this model was used to calculate the difference in allele frequencies between wild individuals from 1925 to 1926 and hatchery-produced fish captured during 1961–1967. To estimate the difference between wild (*n* = 160) and hatchery-produced fish (*n* = 81) in *vgll3* and *six6* allele frequency for the combined 1987 and 2016 data, we used quasi-binomial models with year and fish type as fixed effects.

### Modeling the Adaptive Dynamics.

To quantitatively assess whether adaptation of salmon body size to reduced waterflow was in concordance with evolutionary theory, we developed a statistical model that could be fitted to the data. The model is simple and based on the standard deterministic quantitative genetic model where the mean trait value in the next generation (before selection) is given by ${\bar{z}}^{'}=\bar{z}+V_{A}\beta$, where $\bar{z}$ is the mean trait value for the parents (before selection), $V_{A}$ is the additive genetic variance, and β is the selection gradient (51, 52). However, with overlapping generations and because we wanted to include the effect of allele frequency changes at two loci, the model becomes more complicated. The modeling of $V_{A}$, including the contribution of large-effect genes and their allele frequency changes, is theoretically well funded and should, to a high degree, reflect reality. The model has several naïve aspects, however. It only includes plasticity in its residuals, and hence the trait value before selection ignores the effect of variation in the environment. It has a simple relationship between phenotype and fitness (i.e., natural selection). We assume that all selection happens in adults with a constant stabilizing selection around an optimum trait value that is deterministically determined by waterflow. Hence, we ignore all other forms of selection, including selection on other traits. We wanted to test whether a simple relationship between waterflow and natural selection could explain the evolution of body size in the Eira salmon, and this is what the statistical model is designed to do.

The Atlantic salmon has overlapping generations that complicate the evolutionary dynamics. Assuming that averages of breeding values combine additively, we moved individuals forward in time from the year they were eggs to obtain the mean log mass (before selection) for the individuals returning at year *t* from the weighted average

$${\bar{z}}_{t}=\underset{k}{\sum }w_{k}\left({\bar{z}}_{t-k}+V_{A(t-k)}\beta_{t-k}\right),$$

where the weight $w_{k}$ is the proportion of individuals that were eggs in year t−k, given by 0.114, 0.336, 0.308, 0.181, and 0.061 for k equal to 4, 5, 6, 7, and 8 y, respectively (and zero elsewise). The proportions were based on the age distribution (at return) across all data.

The evolutionary model starts the first year where we have both phenotypic data and waterflow data, at *t* = 1940. To model the trait means before selection in the 8 y before 1940, we used an informative prior, *N*(9.26, 0.14^2^). This prior was based on the average trait value and its annual variation before the first waterflow reduction in 1953. We modeled mass on the natural log scale in units of ln grams.

The selection gradient β was modeled as an increasing function of distance to the optimum θ, given by

$$\beta_{t}=-\exp(q)\left({\bar{z}}_{t}-\theta_{t}\right),$$

where the parameter q gives the strength of stabilizing selection around the optimum, and exp is the natural exponential function (the negative of which gives stabilizing selection). To ensure that selection did not become unreasonably strong (and keeping the genetic load within reasonable limits), we used the prior $q\approx N(-2,1^{2})$. The optimum at year *t* was a linear function of average waterflow from June to September $\bar{x}$ in units of ln(cubic meters per second),

$$\theta_{t}=\theta_{1940}+b\left({\bar{x}}_{t}-{\bar{x}}_{1940}\right),$$

where the intercept $\theta_{1940}$ gives the optimum phenotype in 1940, and the slope b gives the linear change with a change in waterflow from the 1940 value. Both $\theta_{1940}$ and *b* were modeled as fixed effects.

The total additive genetic variance at year *t* is given by the large-effect loci (SNPs) and additional (unknown) small-effect loci: $V_{A(t)}=V_{A(\mathrm{vgll}3,\;t)}+V_{A(\mathrm{six}6,t)}+V_{A(\mathrm{small})}.$ Because *vgll3* and *six6* have sex-specific effects, $V_{A(\mathrm{vgll}3,t)}$ and $V_{A(\mathrm{six}6,t)}$ will have sex-specific components. The combined contribution of female, *F*, and male, *M*, for the *i*th SNP (*vgll3* or *six6*) at year *t* to the additive genetic variance is

$$V_{A(i,t)}=\frac{1}{4}\left(V_{A}{\left[F\right]}_{i,t}+V_{A}{\left[M\right]}_{i,t}+2C_{A}{\left[F,M\right]}_{i,t}\right),$$

where the variance term for sex *j* is

$$V_{A}{\left[j\right]}_{i,t}=2p_{i,t}\left(1-p_{i,t}\right){\left(a_{i,j}+d_{i,j}\left(1-2p_{i,t}\right)\right)}^{2}-\mathrm{Bias},$$

$p_{i,t}$ is the allele frequency at locus *i* in year *t*, $a_{i,j}$ and $d_{i,j}$ are the additive effect and dominance effect, respectively, for locus *i* in sex *j*,

$$\mathrm{Bias}=2p_{i,t}\left(1-p_{i,t}\right)\left(\text{Var}\left[a_{i,j}\right]+{\left(1-2p_{i,t}\right)}^{2}\text{Var}\left[d_{i,j}\right]+2\left(1-2p_{i,t}\right)\text{Cov}[a_{i,j},d_{i,j}]\right),$$

while the covariance term is given by

$$C_{A}{\left[F,M\right]}_{i,t}=2p_{i,t}\left(1-p_{i,t}\right)\left(a_{i,F}+d_{i,F}\left(1-2p_{i,t}\right)\right)\left(a_{i,M}+d_{i,M}\left(1-2p_{i,t}\right)\right)-\mathrm{Bias},$$

where

$$\mathrm{Bias}=2p_{\text{i},t}\left(1-p_{i,t}\right)\left(\text{Cov}\left[a_{i,F},a_{i,M}\right]+\left(1-2p_{i,t}\right)\text{Cov}\left[a_{i,F},d_{i,M}\right]+\left(1-2p_{i,t}\right)\text{Cov}\left[d_{i,F},a_{i,M}\right]+{\left(1-2p_{i,t}\right)}^{2}\text{Cov}\left[d_{i,F},d_{i,M}\right]\right).$$

See *SI Appendix*, Table S3 for the genetic effects and associated error (co)variances used in the model.

To model the genetic variance due to small-effect genes, we used $V_{A(\mathrm{small})}=h^{2}V_{P}-V_{A(\mathrm{vgll}3,t<1940)}-V_{A(\mathrm{six}6,t<1940)}$, where $h^{2}$ is the heritability and $V_{P}$ is the phenotypic variance. We modeled the heritability on logit scale using an informative, but not very strong, prior to ensure reasonable values: $\text{logit}\;h^{2}\approx N\left(0,1^{2}\right)$. The within-year phenotypic variance, $V_{P}$, before the first development was estimated at 0.31 log^2^(g) and used as a constant in the model.

To model changes in allele frequencies due to selection, $\Delta p_{i,t}$, we used the fact that

$$2\Delta p_{i,t}\left(a_{i}+d_{i}\left(1-2p_{i,t}\right)\right)\approx V_{A(i,t)}\beta_{t},$$

where $V_{A(i,t)}\beta_{t}$ is the evolutionary response due to selection at locus *i*. Rearranging and accounting for the sex-specific genetic effects and variances yields

$$\Delta p_{i,t}\approx \frac{\left(V_{A}{\left[F\right]}_{i,t}+C_{A}{\left[F,M\right]}_{i,t}\right)\beta_{t}}{8\left(a_{i,F}+d_{i,F}\left(1-2p_{i,t}\right)\right)}+\frac{\left(V_{A}{\left[M\right]}_{i,t}+C_{A}{\left[F,M\right]}_{i,t}\right)\beta_{t}}{8\left(a_{i,M}+d_{i,M}\left(1-2p_{i,t}\right)\right)}.$$

This approximation is good for small changes in allele frequency or if there is little dominance.

Similarly as for the average trait value, we modeled the allele frequency at time *t* as a weighted average by moving individuals forward in time from when they were eggs until they returned to the river as adults,

$$p_{i,t}=\underset{k}{\sum }w_{k}\left(p_{i,t-k}+\Delta p_{i,t-k}\right).$$

We modeled the parental allele frequencies ($p_{i,t-k}$) before 1940 as one value for each locus using informative priors on the logit scale. These priors were chosen to reflect the observed allele frequencies in 1925–1926, and given by logit *p_vgll3_* ≈ *N*(0.99, 0.20^2^) and logit *p_six6_* ≈ *N*(2.94, 0.80^2^).

To fit the model to data on average observed log mass at each year *t*, ${\bar{z}}_{\mathrm{obs}(t)}$, we used the observation model

$${\bar{z}}_{\mathrm{obs}(t)}=\;{\bar{z}}_{t}+\delta s_{t}+e_{t}+m_{t},$$

where ${\bar{z}}_{t}$ is the trait mean before selection, δ is the fraction of selection happening before we observed the trait mean (estimated as a fixed effect on the logit scale), $s_{t}=V_{P}\beta_{t}$ is the selection differential, $e_{t}\approx N\left(0,\sigma_{e}^{2}\right)$ is the residual deviation from the model, and $m_{t}\approx N\left(0,SE_{t}^{2}\right)$ is the measurement error, where *SE_t_* is the standard error of ${\bar{z}}_{\mathrm{obs}(t)}$.

For the allele frequencies estimates of 1987 and 2016, we used the observation model

$$A_{i,t\;}∼\mathrm{Bin}\;\left(p_{i,t}+\delta \Delta p_{i,t},\;T_{i}\right),$$

where *A* is the number of observed alleles of one type, and T is the total number of observed alleles.

The data on observed trait means and allele frequencies inform the parameters *q*, $\theta_{1940}$, *b*, δ, *h^2^*, and $\sigma_{e}^{2}$, in addition to the initial (*t* < 1940) trait values and allele frequencies. We used R (53) and the Template Model Builder package, TMB (54), which fits the model to the data using maximum likelihood.

## Supplementary Material

Supplementary File

## Data Availability

Waterflow data, genotypes and metadata for all individuals that scale samples exist for, metadata for all individuals included in the study, and computer code have been deposited in Zenodo (10.5281/zenodo.7049816) (55).

## References

[1] G. Ceballos, P. R. Ehrlich, R. Dirzo, Biological annihilation via the ongoing sixth mass extinction signaled by vertebrate population losses and declines. Proc. Natl. Acad. Sci. U.S.A. 114, E6089–E6096 (2017). 28696295 10.1073/pnas.1704949114 PMC5544311

[2] G. Bell, Evolutionary rescue. Annu. Rev. Ecol. Evol. Syst. 48, 605–627 (2017).

[3] S. Sanderson , The pace of modern life, revisited. Mol. Ecol. 31, 1028–1043 (2022). 34902193 10.1111/mec.16299

[4] L. M. Cook, I. J. Saccheri, The peppered moth and industrial melanism: Evolution of a natural selection case study. Heredity 110, 207–212 (2013). 23211788 10.1038/hdy.2012.92 PMC3668657

[5] Y. Czorlich, T. Aykanat, J. Erkinaro, P. Orell, C. R. Primmer, Rapid evolution in salmon life history induced by direct and indirect effects of fishing. Science 376, 420–423 (2022). 35201899 10.1126/science.abg5980

[6] T. Q. Thompson , Anthropogenic habitat alteration leads to rapid loss of adaptive variation and restoration potential in wild salmon populations. Proc. Natl. Acad. Sci. U.S.A. 116, 177–186 (2019). 30514813 10.1073/pnas.1811559115 PMC6320526

[7] S. C. Campbell-Staton , Ivory poaching and the rapid evolution of tusklessness in African elephants. Science 374, 483–487 (2021). 34672738 10.1126/science.abe7389

[8] F. Ayllon , The *vgll3* locus controls age at maturity in wild and domesticated Atlantic salmon (*Salmo salar* L.) males. PLoS Genet. 11, e1005628 (2015). 26551894 10.1371/journal.pgen.1005628 PMC4638356

[9] N. J. Barson , Sex-dependent dominance at a single locus maintains variation in age at maturity in salmon. Nature 528, 405–408 (2015). 26536110 10.1038/nature16062

[10] M. Sinclair-Waters , Beyond large-effect loci: Large-scale GWAS reveals a mixed large-effect and polygenic architecture for age at maturity of Atlantic salmon. Genet. Sel. Evol. 52, 9 (2020). 32050893 10.1186/s12711-020-0529-8 PMC7017552

[11] E. B. Thorstad, F. Whoriskey, A. H. Rikardsen, K. Aarestrup, “Aquatic nomads: The life and migrations of the Atlantic salmon” in Atlantic Salmon Ecology, Ø. Aas, S. Einum, A. Klemetsen, J. Skurdal, Eds. (Blackwell, Oxford, UK, 2011), pp. 1–32.

[12] I. A. Fleming, Pattern and variability in the breeding system of Atlantic salmon (*Salmo salar*), with comparisons to other salmonids. Can. J. Fish. Aquat. Sci. 55, 59–76 (1998).

[13] G. Power, Stock characteristics and catches of Atlantic salmon (*Salmo salar*) in Quebec, and Newfoundland and Labrador in relation to environmental variables. Can. J. Fish. Aquat. Sci. 38, 1601–1611 (1981).

[14] N. Jonsson, L. P. Hansen, B. Jonsson, Variation in age, size and repeat spawning of adult Atlantic salmon in relation to river discharge. J. Anim. Ecol. 60, 937–947 (1991).

[15] C. Nilsson, C. A. Reidy, M. Dynesius, C. Revenga, Fragmentation and flow regulation of the world’s large river systems. Science 308, 405–408 (2005). 15831757 10.1126/science.1107887

[16] B. Belletti , More than one million barriers fragment Europe’s rivers. Nature 588, 436–441 (2020). 33328667 10.1038/s41586-020-3005-2

[17] J. A. Hutchings, M. E. B. Jones, Life history variation and growth rate thresholds for maturity in Atlantic salmon, *Salmo salar*. Can. J. Fish. Aquat. Sci. 55, 22–47 (1998).

[18] G. Bremset , Fiskebiologiske undersøkelser i Auravassdraget. Sluttrapport fra undersøkelsene i perioden 2014-2018 (NINA Rapport 1583, Norwegian Institute for Nature Research, 2019).

[19] S. Karlsson, T. Moen, S. Lien, K. A. Glover, K. Hindar, Generic genetic differences between farmed and wild Atlantic salmon identified from a 7K SNP-chip. Mol. Ecol. Resour. 11, 247–253 (2011). 21429178 10.1111/j.1755-0998.2010.02959.x

[20] E. Cauwelier, J. Gilbey, J. Sampayo, L. Stradmeyer, S. J. Middlemas, Identification of a single genomic region associated with seasonal river return timing in adult Scottish Atlantic salmon (*Salmo salar*), using a genome-wide association study. Can. J. Fish. Aquat. Sci. 75, 1427–1435 (2017).

[21] V. L. Pritchard , Genomic signatures of fine-scale local selection in Atlantic salmon suggest involvement of sexual maturation, energy homeostasis and immune defence-related genes. Mol. Ecol. 27, 2560–2575 (2018). 29691916 10.1111/mec.14705

[22] J. Hereford, T. F. Hansen, D. Houle, Comparing strengths of directional selection: How strong is strong? Evolution 58, 2133–2143 (2004). 15562680 10.1111/j.0014-3820.2004.tb01592.x

[23] T. F. Hansen, C. Pélabon, W. S. Armbruster, M. L. Carlson, Evolvability and genetic constraint in Dalechampia blossoms: Components of variance and measures of evolvability. J. Evol. Biol. 16, 754–766 (2003). 14632238 10.1046/j.1420-9101.2003.00556.x

[24] M. Tarka , Did natural selection make the Dutch taller? A cautionary note on the importance of quantification in understanding evolution. Evolution 69, 3204–3206 (2015). 26507881 10.1111/evo.12803

[25] J. C. Uyeda, T. F. Hansen, S. J. Arnold, J. Pienaar, The million-year wait for macroevolutionary bursts. Proc. Natl. Acad. Sci. U.S.A. 108, 15908–15913 (2011). 21873251 10.1073/pnas.1014503108 PMC3179053

[26] R. Gomulkiewicz, D. Houle, Demographic and genetic constraints on evolution. Am. Nat. 174, E218–E229 (2009). 19821744 10.1086/645086

[27] D. Jablonski, Extinction: Past and present. Nature 427, 589–589 (2004). 14961099 10.1038/427589a

[28] A. Le Rouzic, D. Houle, T. F. Hansen, A modelling framework for the analysis of artificial-selection time series. Genet. Res. 93, 155–173 (2011). 10.1017/S0016672311000024 21473802

[29] B. Walsh, M. W. Blows, Abundant genetic variation + strong selection = multivariate genetic constraints: A geometric view of adaptation. Annu. Rev. Ecol. Evol. Syst. 40, 41–59 (2009).

[30] K. W. Vollset , Ecological regime shift in the Northeast Atlantic Ocean revealed from the unprecedented reduction in marine growth of Atlantic salmon. Sci. Adv. 8, eabk2542 (2022). 35245115 10.1126/sciadv.abk2542 PMC8896796

[31] I. J. Hagen , Evaluation of genetic effects on wild salmon populations from stock enhancement. ICES J. Mar. Sci. 78, 900–909 (2020).

[32] N. Ryman, L. Laikre, Effects of supportive breeding on the genetically effective population size. Conserv. Biol. 5, 325–329 (1991).

[33] D. E. Schindler , Population diversity and the portfolio effect in an exploited species. Nature 465, 609–612 (2010). 20520713 10.1038/nature09060

[34] A. J. Jensen , Fiskebiologiske undersøkelser i Auravassdraget. Årsrapport for 2015 (NINA Rapport 1249, Norwegian Institute for Nature Research, 2016).

[35] A. J. Jensen , Passing a seawater challenge test is not indicative of hatchery-reared Atlantic salmon *Salmo salar* smolts performing as well at sea as their naturally produced conspecifics. J. Fish Biol. 88, 2219–2235 (2016). 27133912 10.1111/jfb.12984

[36] B. Carlin, Tagging of salmon smolts in the river Lagan. Rep. Inst. Freshwater Res. Drottningholm 36, 57–74 (1955).

[37] A. J. Jensen , Synchrony in marine growth among Atlantic salmon (*Salmo salar*) populations. Can. J. Fish. Aquat. Sci. 68, 444–457 (2011).

[38] B. S. Weir, C. C. Cockerham, Estimating F-statistics for the analysis of population structure. Evolution 38, 1358–1370 (1984). 28563791 10.1111/j.1558-5646.1984.tb05657.x

[39] P. Fiske, R. A. Lund, L. P. Hansen, “Identifying fish farm escapees” in Stock Identification Methods, S. X. Cadrin, K. D. Friedland, J. R. Waldman, Eds. (Elsevier, Amsterdam, Netherlands, 2005), chap. 31, pp. 659–680.

[40] E. Quéméré , An improved PCR-based method for faster sex determination in brown trout (*Salmo trutta*) and Atlantic salmon (*Salmo salar*). Conserv. Genet. Resour. 6, 825–827 (2014).

[41] S. Karlsson , A genetic marker for the maternal identification of Atlantic salmon × brown trout hybrids. Conserv. Genet. Resour. 5, 47–49 (2013).

[42] S. Karlsson, O. H. Diserud, T. Moen, K. Hindar, A standardized method for quantifying unidirectional genetic introgression. Ecol. Evol. 4, 3256–3263 (2014). 25473478 10.1002/ece3.1169 PMC4222212

[43] S. Karlsson, O. H. Diserud, P. Fiske, K. Hindar, Widespread genetic introgression of escaped farmed Atlantic salmon in wild salmon populations. ICES J. Mar. Sci. 73, 2488–2498 (2016).

[44] R. A. Fisher, The Genetical Theory of Natural Selection (Oxford University Press, Oxford, UK, 1930).

[45] S. Wright, Evolution in Mendelian populations. Genetics 16, 97–159 (1931). 17246615 10.1093/genetics/16.2.97 PMC1201091

[46] P. Tataru, M. Simonsen, T. Bataillon, A. Hobolth, Statistical inference in the Wright-Fisher model using allele frequency data. Syst. Biol. 66, e30–e46 (2017). 28173553 10.1093/sysbio/syw056 PMC5837693

[47] Y. Czorlich, T. Aykanat, J. Erkinaro, P. Orell, C. R. Primmer, Rapid sex-specific evolution of age at maturity is shaped by genetic architecture in Atlantic salmon. Nat. Ecol. Evol. 2, 1800–1807 (2018). 30275465 10.1038/s41559-018-0681-5 PMC6322654

[48] M. Plummer, JAGS version 4.3. 0 user manual. https://sourceforge.net/projects/mcmc-jags/files/Manuals/4.x/. Accessed 30 March 2022.

[49] S. P. Brooks, A. Gelman, General methods for monitoring convergence of iterative simulations. J. Comput. Graph. Stat. 7, 434–455 (1998).

[50] J. M. Álvarez-Castro, O. Carlborg, A unified model for functional and statistical epistasis and its application in quantitative trait loci analysis. Genetics 176, 1151–1167 (2007). 17409082 10.1534/genetics.106.067348 PMC1894581

[51] R. Lande, Quantitative genetic analysis of multivariate evolution, applied to brain: Body size allometry. Evolution 33, 402–416 (1979). 28568194 10.1111/j.1558-5646.1979.tb04694.x

[52] R. Lande, S. J. Arnold, The measurement of selection on correlated characters. Evolution 37, 1210–1226 (1983). 28556011 10.1111/j.1558-5646.1983.tb00236.x

[53] R. Development Core Team, R: A Language and Environment for Statistical Computing (R Foundation for Statistical Computing, Vienna, Austria, 2021).

[54] K. Kristensen, A. Nielsen, C. W. Berg, H. Skaug, B. M. Bell, TMB: Automatic differentiation and Laplace approximation. J. Stat. Softw. 70, 1–21 (2016).

[55] I. J. Hagen ., Large effect loci mediate rapid adaptation of salmon body size after river regulation. Zenodo. https://zenodo.org/record/7049816#.Yz8ilExByUk. Deposited 8 October 2022. 10.1073/pnas.2207634119 PMC9636922 36279467

