# Supplementary material for: Large-effect loci mediate rapid adaptation of salmon body size after river regulation
Source: Proc Natl Acad Sci U S A. 2022 Oct 24;119(44):e2207634119. doi: 10.1073/pnas.2207634119 (PMC9636922; doi:10.1073/pnas.2207634119)
Supplement: Supplementary File [file pnas.2207634119.sapp.pdf]

PNAS

Supplementary Information for

Large effect loci mediate rapid adaptation of salmon body size after river regulation

Arne J. Jensen, Ingerid J. Hagen, Yann Czorlich, Geir H. Bolstad, Gunnbjørn Bremset, Bengt Finstad, Kjetil Hindar, Øystein Skaala and Sten Karlsson

Ingerid J. Hagen; Sten Karlsson

Email: ingerid.hagen@nina.no; sten.karlsson@nina.no

This PDF file includes:

- Figures S1 to S15
- Tables S1 to S6
- Legends for Datasets

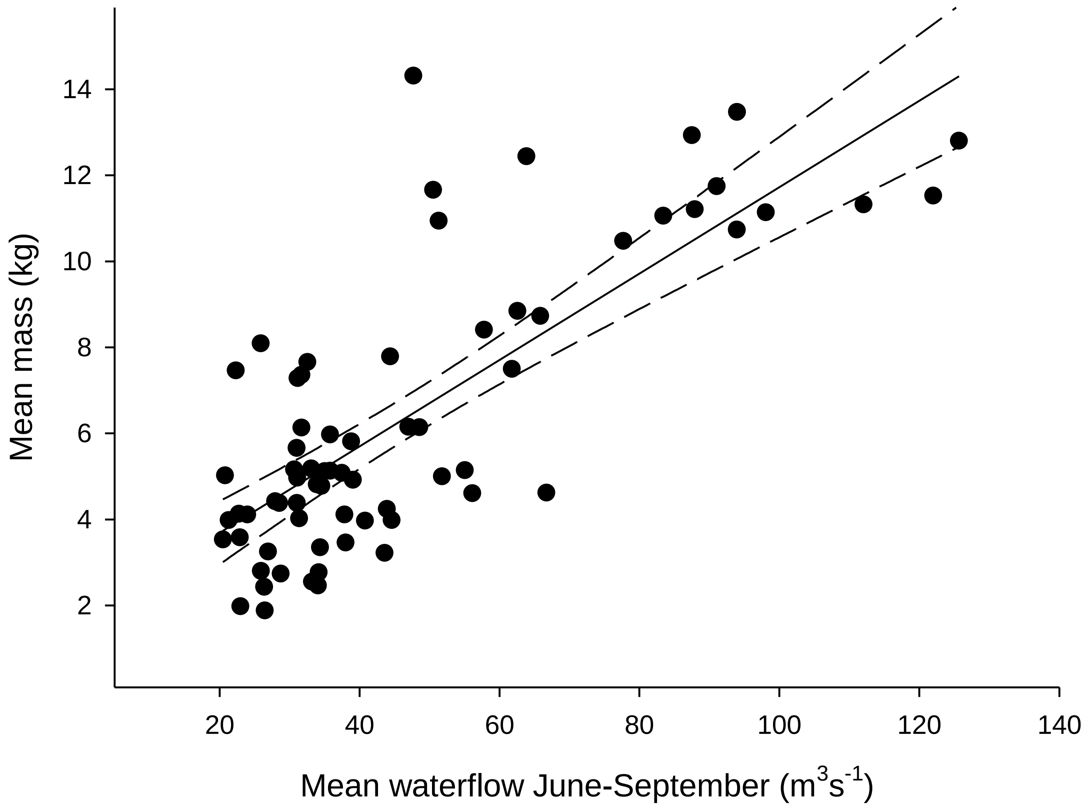

Fig. S1. Waterflow and body mass of Atlantic salmon in river Eira. Relationship between mean waterflow during the upward migration period (June - September) and mean body mass in catches of Atlantic salmon ($y = 1.637\ (\pm 0.53) + 0.100\ (\pm 0.01)\ x$, $r^2 = 0.599$).

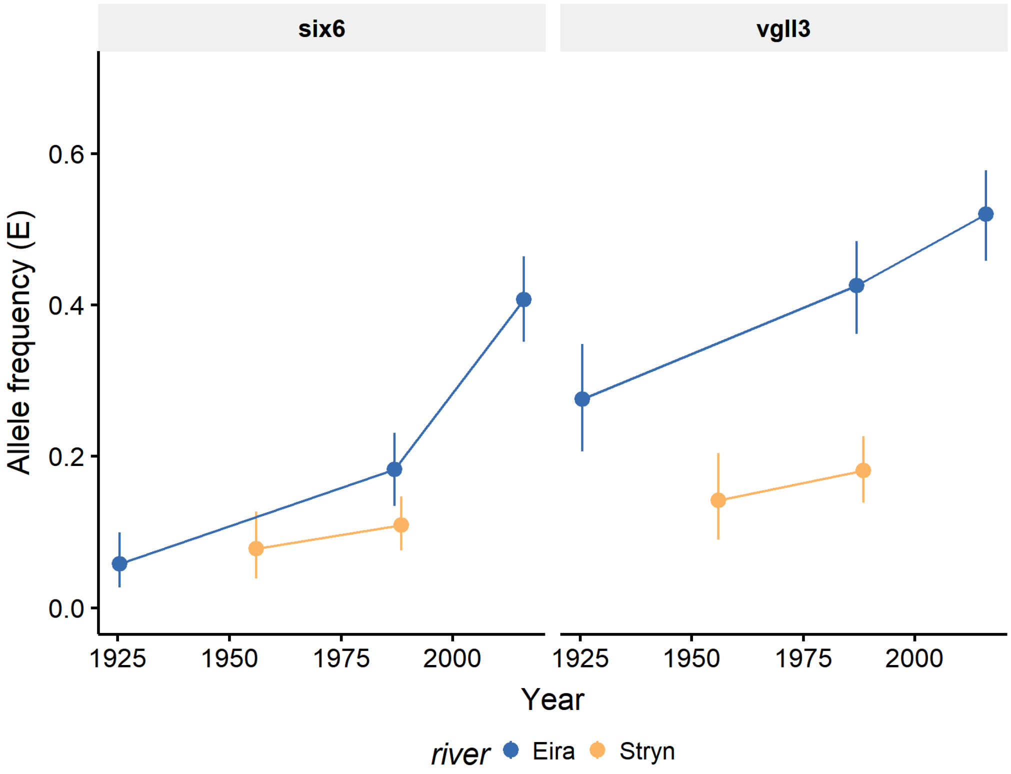

Fig. S2. Allele frequencies for the *E* alleles for *vgll3* and *six6* in Rivers Eira and Stryn. The *E* alleles are associated with small body size. Vertical bars represent 95% credible interval.

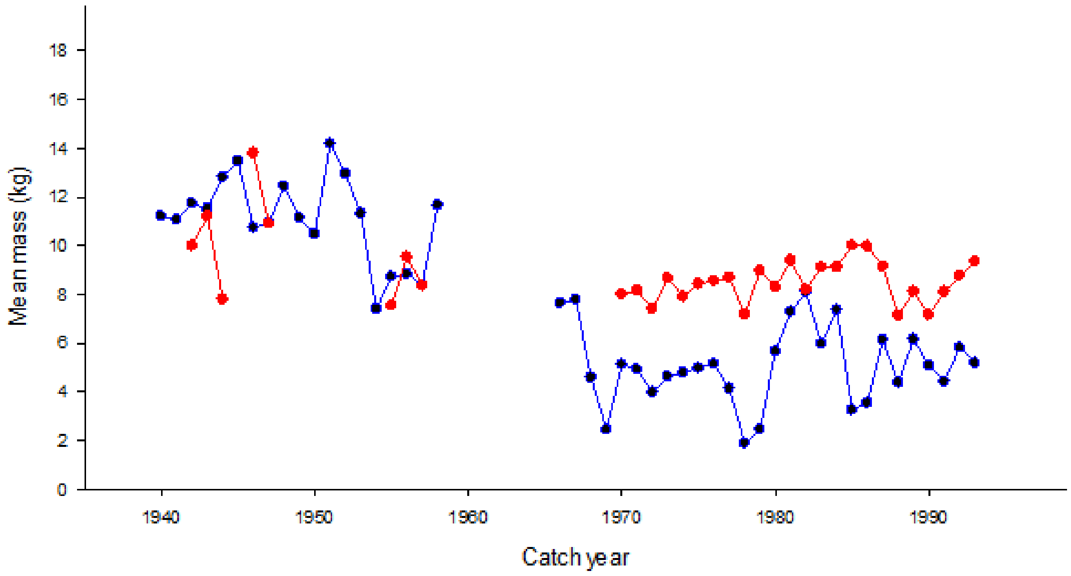

Fig. S3. Mean body mass of Atlantic salmon caught in Rivers Eira and Stryn. Mean body mass of salmon caught in River Eira (blue) and River Stryn (red) during the angling season in the period 1940-1993.

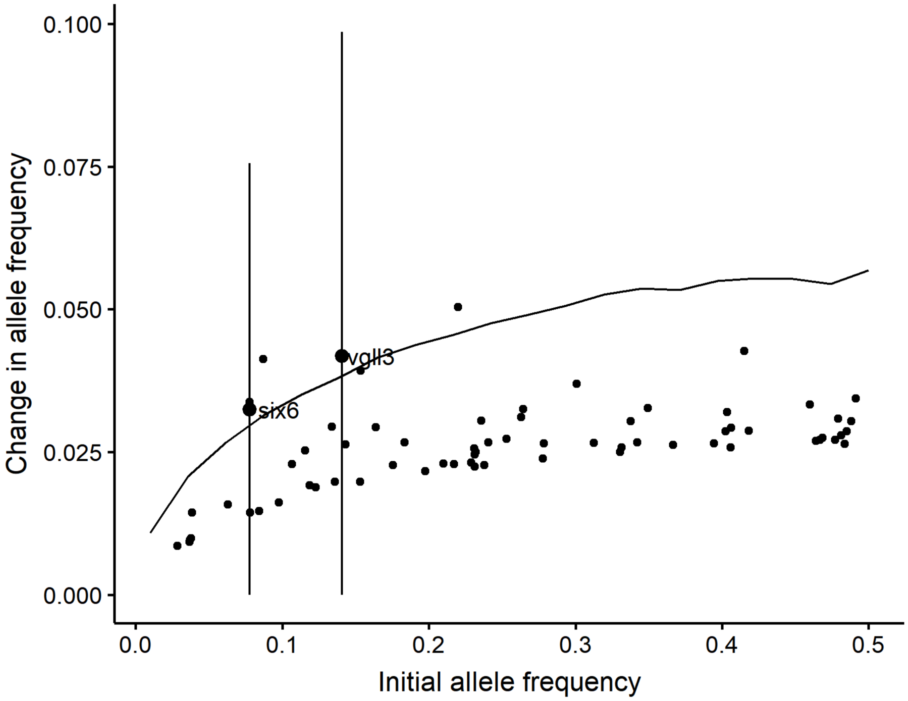

Fig. S4. Absolute allele frequency changes for the genes *vgll3* and *six6* from 1956/1957-1990 in Atlantic salmon from River Stryn in comparison with putatively neutral markers. Each dot represents a SNP-marker. The genes *vgll3* and *six6* are indicated. The remaining 67 neutral markers represent genetic drift. Vertical bars represent 95% credible intervals. The solid line represents the absolute amount of change expected under drift at the 95 quantiles.

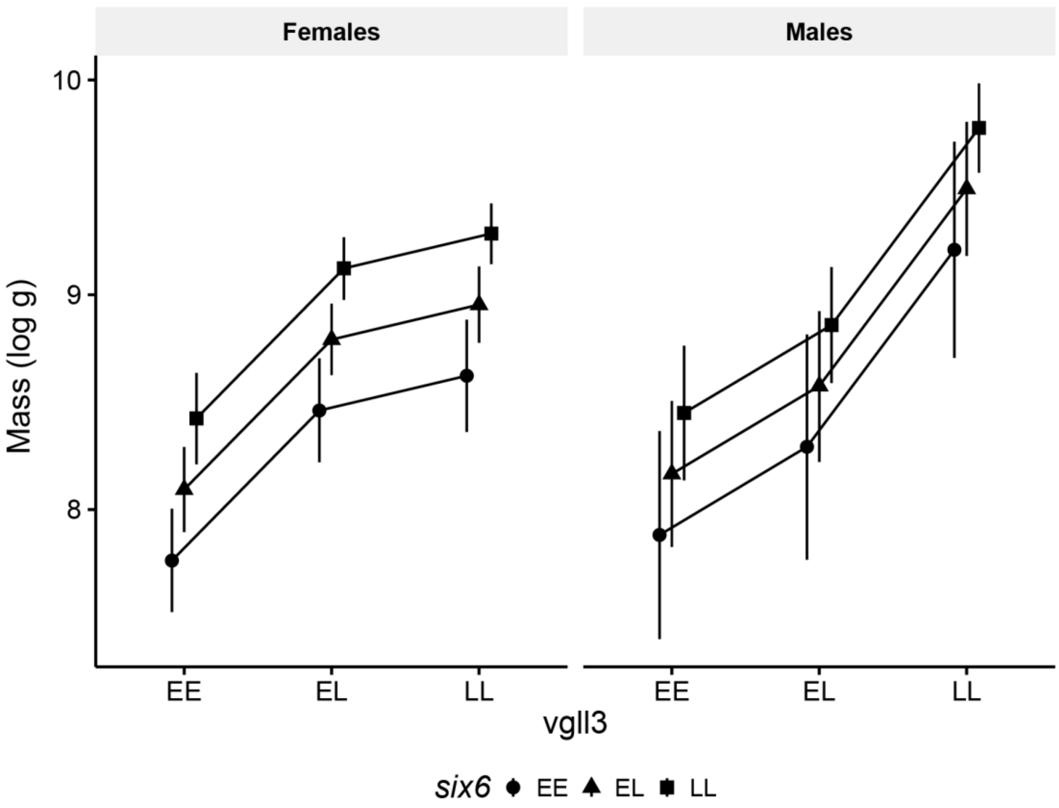

Fig. S5. Functional effect of *vgll3* and *six6* on body mass in females and males. The error bars correspond to 95% confidence intervals. The years of reference for the intercept are 1925-1926. See Table S3 for parameter estimates.

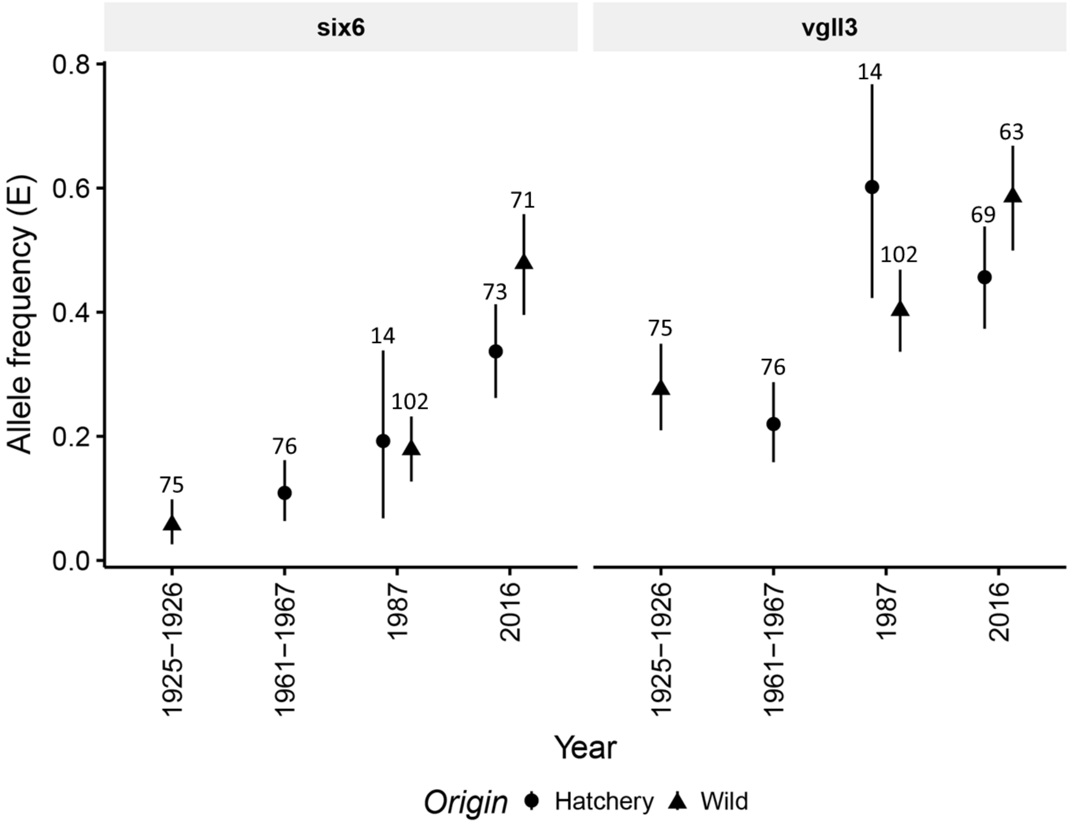

Fig. S6. Allele frequencies for *six6* and *vgll3* in naturally produced individuals and hatchery-released individuals. Numbers in the figure represent the number of individuals for which the respective SNP markers were successfully genotyped. Vertical bars correspond to 95% confidence intervals.

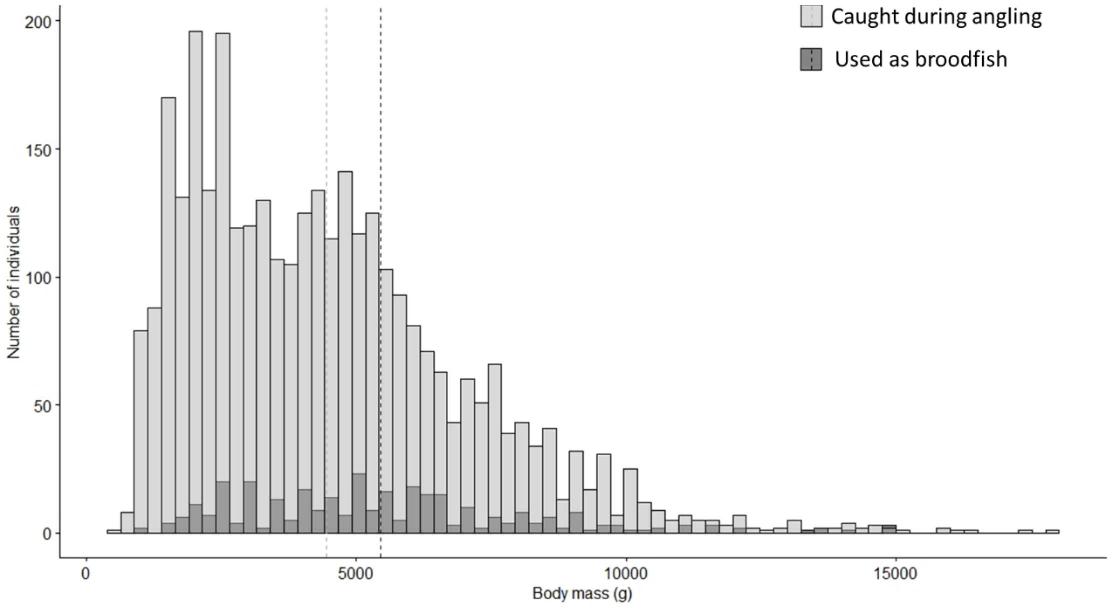

Fig. S7: The body mass of River Eira broodfish compared to fish caught and culled during the angling season for the run years 2005-2016. The broken lines indicate the averages for the two data series. It is unlikely that broodfish smaller than the population average was used during the early years of stocking.

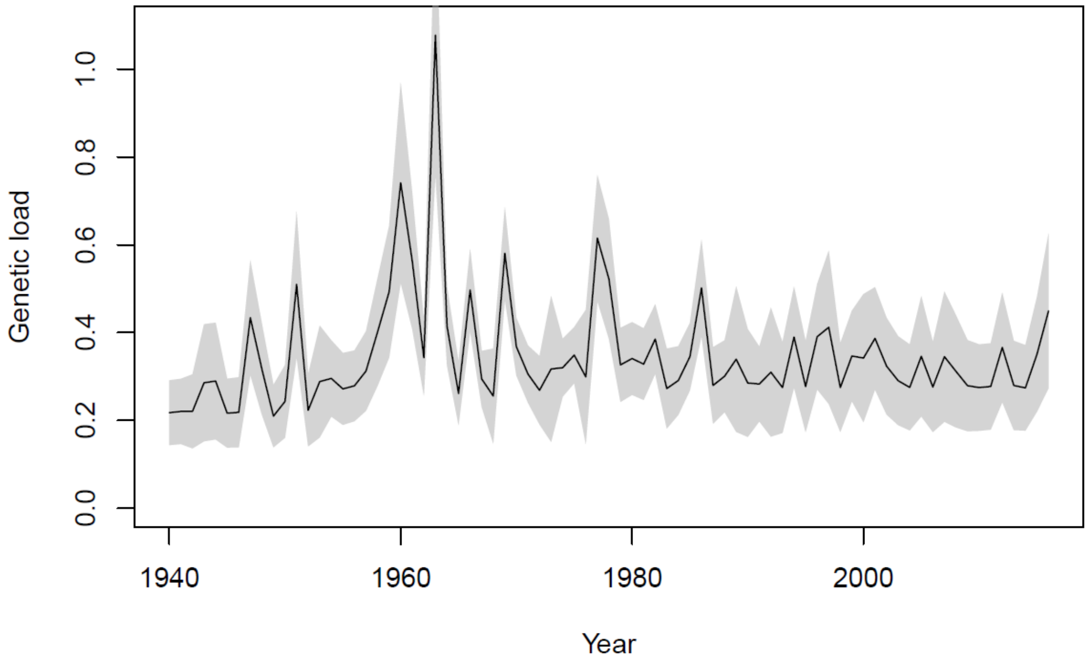

Fig. S8. Genetic load. Genetic load estimated by the adaptive dynamics model. The genetic load, L, is defined as $(W_\theta - \overline{W})/W_\theta$, where W_θ is the fitness at the optimum and $\overline{W}$ is the average fitness of the population. In our model this can be estimated as $L_t = -\frac{1}{2}\exp(q)\left((\overline{z}_t - \theta_t)^2 + (1 - h^2)V_p + V_{A(t)}\right)$. Shaded area indicates ± one standard error.

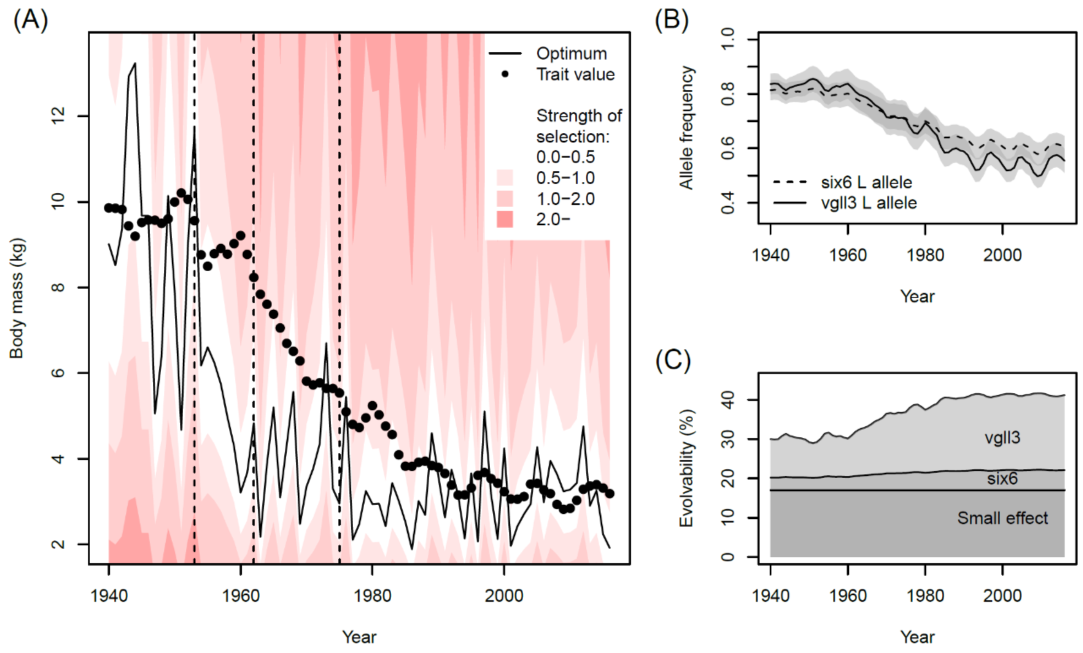

Fig. S9. Adaptive dynamics with long generation time. Assuming that the proportion of parents in year t-k was given by 0.094, 0.345, 0.328, 0.186 and 0.049 for k equal to 6, 7, 8, 9 and 10 years, respectively (and zero elsewise), and ensuring that selection was the same as in the original model by using a very strong prior: $q \sim N(0.32, 0.1^2)$, increased the number of years before adaptation. See Fig. 3 for comparison and explanation of symbols and panels.

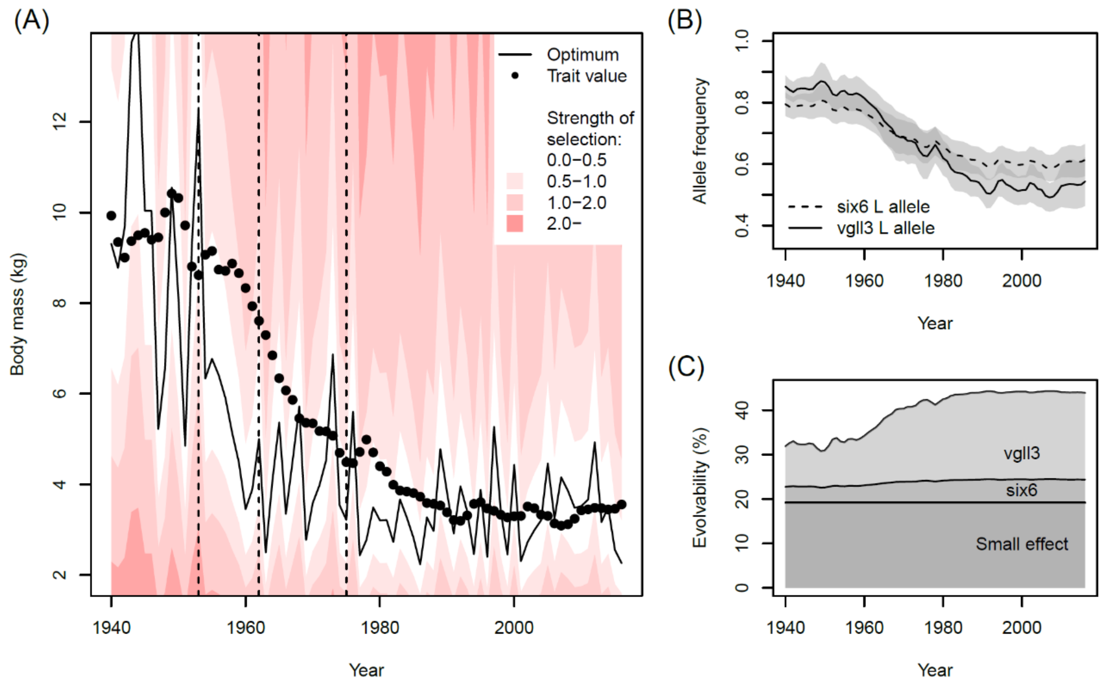

Fig. S10. Adaptive dynamics with a non-linear scaling relationship between waterflow and optimum. Including a quadratic term in the relationship between optimum and waterflow, $\theta_t = \theta_{1940} + b_1(\bar{x}_t - \bar{x}_{1940}) + b_2(\bar{x}_t - \bar{x}_{1940})^2$, had little effect on the adaptive dynamics. The quadratic term was small and uncertain: $b_2 = 0.10 \pm 0.26$. See Fig. 3 for comparison and explanation of symbols and panels.

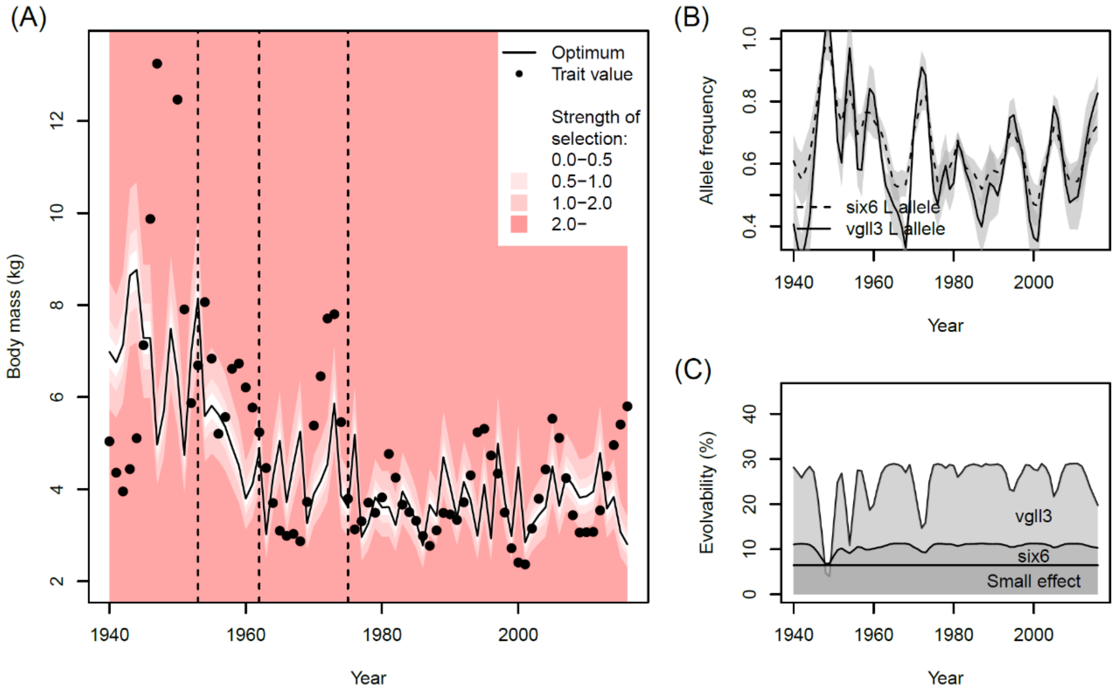

Fig. S11. Adaptive dynamics with a weaker prior on strength of stabilizing selection. Using a weaker prior on q, $N(-2,1.5^2)$, gave very strong selection and unreasonable dynamics. See Fig. 3 for comparison and explanation of symbols and panels.

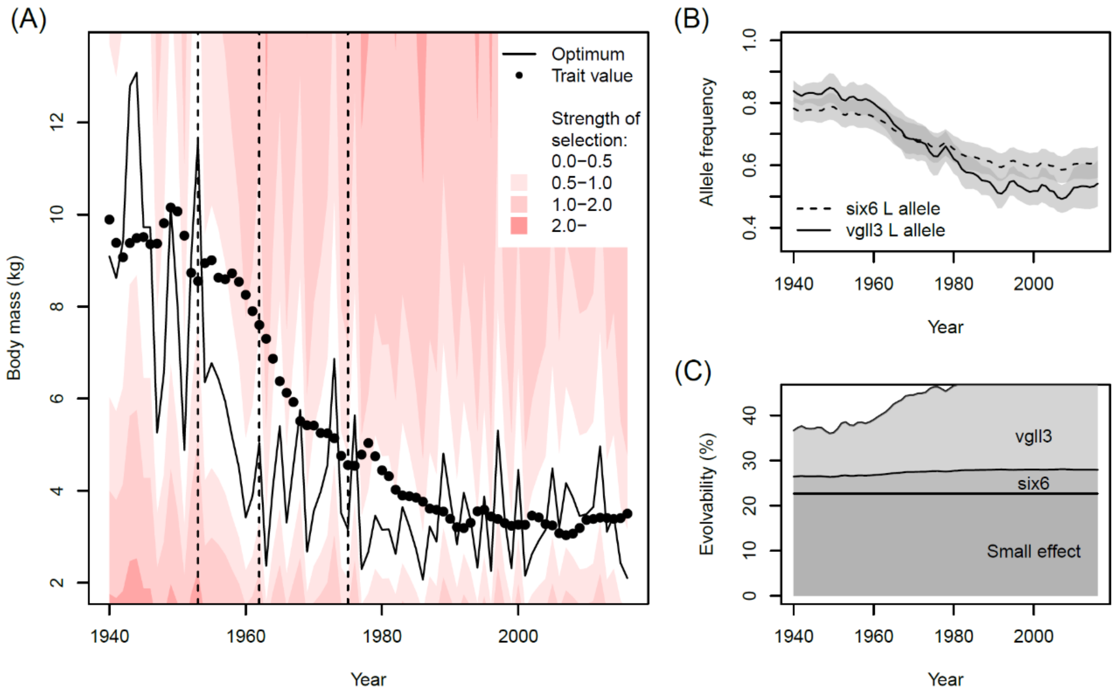

Fig. S12. Adaptive dynamics with no prior on heritability. Modelling logit h^2 as a fixed effect had little effect on the evolutionary dynamics, but selection was weaker and evolvability higher. However, the heritability was estimated to be unreasonably high with logit h^2 = 23±20637, corresponding to a heritability of 1.00. The original model had heritability estimated at 0.89 (Table S4), which is already very high. See Fig. 3 for comparison and explanation of symbols and panels.

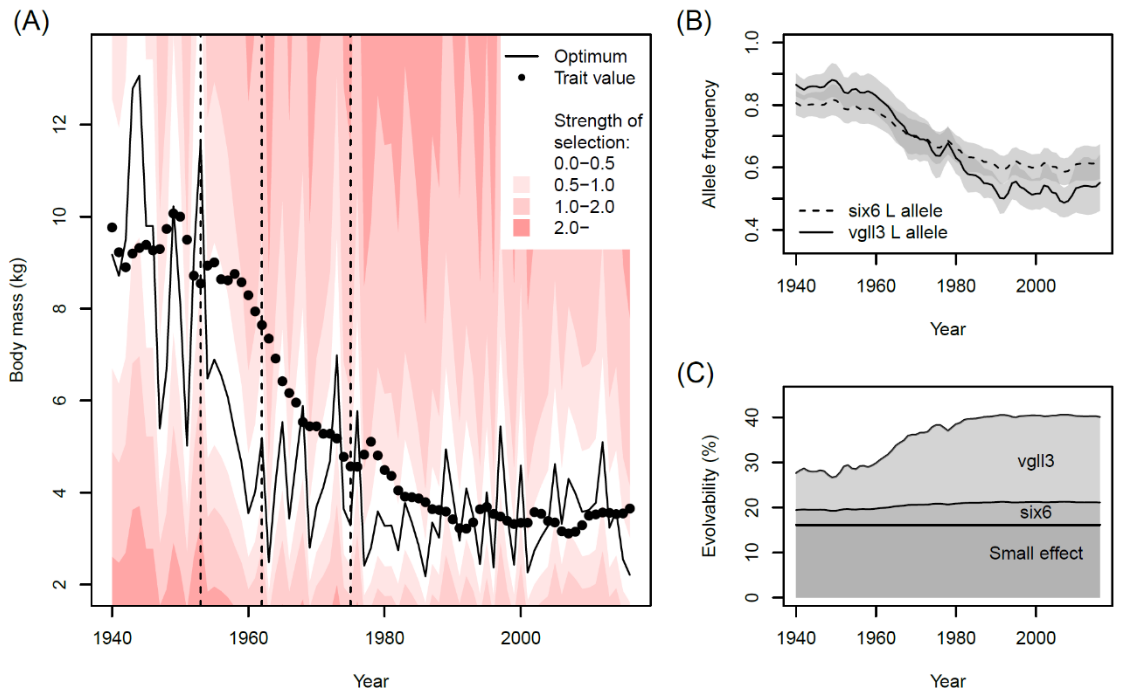

Fig. S13. Adaptive dynamics with a stronger prior towards intermediate heritability. Using a stronger prior on logit h^2, $N(0,0.5^2)$, gave somewhat lower evolvability and stronger selection. In this model the parameter estimate of logit h^2 was estimated at 1.29±0.32, corresponding to a heritability of 0.78. See Fig. 3 for comparison and explanation of symbols and panels.

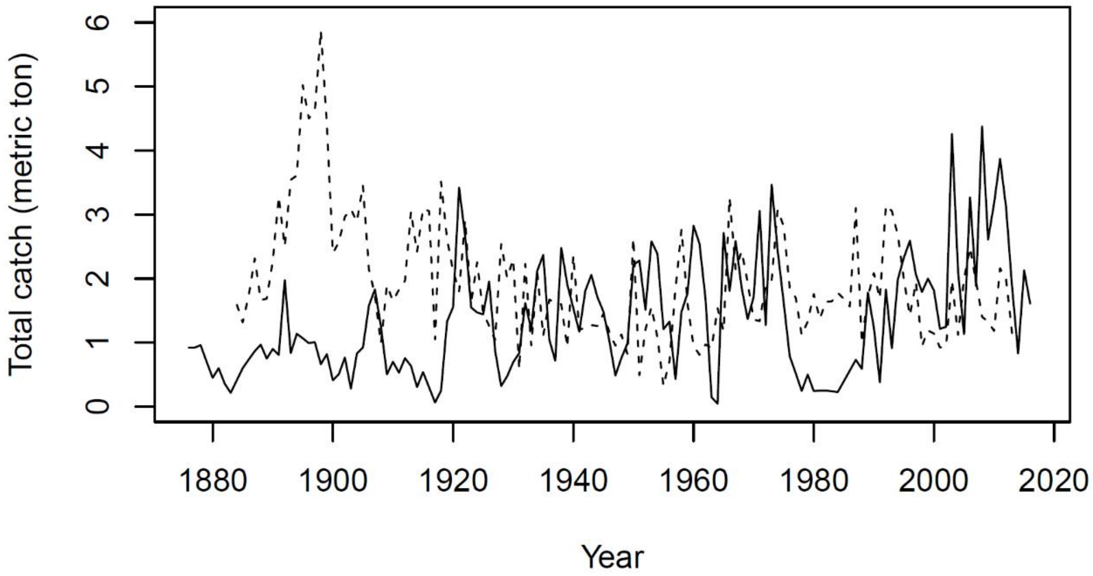

Fig. S14. Official catch statistics for Rivers Eira and Stryn. Total reported catch of salmon (*Salmo salar*) and sea trout (*S. trutta*) in Eira (solid line) and Stryn (dashed line). Note that the catch in these rivers is dominated by salmon. The data is available from the database "Lakseregisteret" of the Norwegian Environmental Agency, https://lakseregisteret.fylkesmannen.no/.

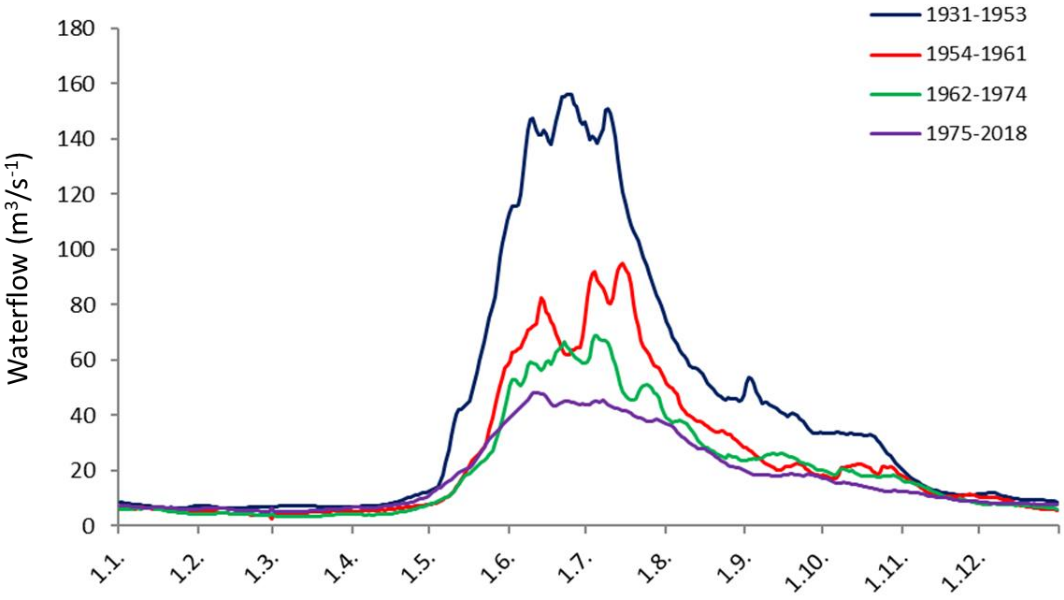

Fig. S15. Mean waterflow in River Eira. Mean monthly waterflow before the first hydropower development (1931-1953), after the first development (1954-1961), after the second development (1962-1974), and after the third development (1975-present). Data are from the Norwegian Water Resources and Energy Directorate.

Table S1. Mean body mass of Atlantic salmon caught by rod in River Eira. Mean body mass (kg), mean smolt age (yr) and mean sea age (yr) of Atlantic salmon in catches before the first hydropower development (1925-1926 and 1940-1953), and after the first (1954-1957), second (1966-1974), and third (1975-2016) development. Smolt ages and sea ages were only available for 1925-1926 and 1987-2016. Means are given with ± their standard error and sample size, *n*, in parenthesis.

Period	*Mass (n)*	Smolt age (*n*)	*Sea age* (*n*)
1925-1926	10.43±0.37 (84)	3.99±0.07 (75)	2.81±0.09 (78)
1940-1953	11.80±0.11 (900)		
1954-1957	8.22±0.25 (190)		
1966-1974	4.83±0.10 (1229)		
1975-2016	4.35±0.04 (5921)	2.89±0.01 (2550)	1.80±0.01 (4947)

Table S2. Effects of age composition and mass within age on mean body mass. Difference in mean body mass ± SE between the periods 1925-1926 and 1987-2016 controlling or not for sea age in the analysis.

Model	Difference*
No control	-6.02±0.31 kg
Controlling for sea age	-3.28±0.18 kg

* The difference was estimated in a linear mixed model with period (1925-1926 and 1987-2016) as a fixed effect and controlling or not controlling for sea age as a fixed factor.

Table S3. Parameter estimates and associated error variance matrix for the model of additive (a) and dominance (d) of *vgll3* and *six6* for log mass (ln g) in females (F) and males (M). Additive and dominance effects were centered within period (Period 2 = 1987; Period 3 = 2016).

Parameter*	Est	Error (co)variance matrix (100x)§											
Int	9.11	0.35	-0.35	0.00	0.00	0.00	0.00	0.00	0.00	-0.35	0.00	-0.35	0.00
Contrast M	0.33		1.33	0.00	0.00	0.00	0.00	0.00	0.00	0.35	-0.97	0.35	-0.97
$d_{vgll3,F}$	0.27			0.59	0.00	0.09	0.00	-0.04	0.00	0.00	0.00	0.00	0.00
$d_{vgll3,M}$	-0.25				1.22	0.00	0.04	0.00	-0.20	0.00	0.00	0.00	0.00
$a_{vgll3,F}$	0.43					0.25	0.00	-0.11	0.00	0.00	0.00	-0.01	0.00
$a_{vgll3,M}$	0.66						0.61	0.00	-0.24	0.00	0.00	0.00	-0.01
$a_{six6,F}$	0.33							0.32	0.00	0.00	0.00	0.01	0.00
$a_{six6,M}$	0.28								1.30	0.00	0.00	0.00	0.03
Contrast Period 2 F	-0.55									0.64	0.00	0.35	0.00
Contrast Period 2 M	-1.32										1.53	0.00	0.97
Contrast Period 3 F	-0.68											0.77	0.00
Contrast Period 3 M	-1.11												2.06

* ”Int” is the intercept reflecting the females of the average genotype in period 1. “Contrast M” is the contrast to the males in period 1, and further contrasts are given for females and males in period 2 and 3.

§ Values in the error variance matrix are multiplied by 100. The variance matrix is symmetric. The lower triangle is deleted for readability.

Table S4. Parameter estimates of the adaptive dynamics model.

Parameter*	Estimate	SE	Units
q	0.32	0.36	$\ln[\ln^{-2}(g)]$
θ_{1940}	9.12	0.10	ln(g)
b	1.00	0.11	$\ln(g)/\ln(m^3 s^{-1})$
logit δ	19	8529	log odds
logit h^2	2.11	0.55	log odds
ln σ_e	-1.16	0.96	$\ln[\ln^2(g)]$

* q is the strength of stabilizing selection, θ_{1940} is the optimum trait value in 1940, b is the slope of the relationship between waterflow and the optimal trait value, δ is the proportion of selection happening before the observations, h^2 is the narrow sense heritability, and σ_e is the residual standard deviation.

Table S5. Output from the adaptive-dynamics model including the genetic value of body mass before selection ($\overline{z}$), optimal body size (θ), *vgll3* L allele frequency (p_{vgll3}), six6 L allele frequency (p_{six6}), additive genetic variance (V_A), vgll3 contribution to additive genetic variance ($V_{A(vgll3)}$), six6 contribution to additive genetic variance ($V_{A(six6)}$), selection gradient (β), selection differential (s), genetic load (L). Estimates are given with ± standard error and units in parenthesis.

Year	$\overline{z}$ (ln g)	θ (ln g)	p_{vgll3}	p_{six6}	V_A (ln^2 g)	$V_{A(vgll3)}$ (ln^2 g)	$V_{A(six6)}$ (ln^2 g)	β (ln^{-1} g)	s (ln g)	L
1940	9.19±0.06	9.12±0.10	0.85±0.02	0.79±0.02	0.278±0.018	0.072±0.007	0.029±0.029	-0.10±0.10	-0.03±0.03	0.22±0.07
1941	9.14±0.05	9.07±0.10	0.84±0.02	0.79±0.02	0.283±0.017	0.076±0.007	0.030±0.030	-0.10±0.11	-0.03±0.03	0.22±0.07
1942	9.10±0.05	9.15±0.11	0.84±0.02	0.78±0.02	0.286±0.017	0.079±0.006	0.030±0.030	0.07±0.16	0.02±0.05	0.22±0.08
1943	9.14±0.05	9.46±0.14	0.84±0.02	0.79±0.02	0.282±0.017	0.075±0.007	0.030±0.030	0.44±0.28	0.12±0.08	0.29±0.13
1944	9.15±0.05	9.48±0.14	0.85±0.02	0.79±0.02	0.281±0.018	0.075±0.007	0.030±0.030	0.45±0.27	0.13±0.07	0.29±0.13
1945	9.15±0.05	9.19±0.11	0.84±0.02	0.79±0.02	0.281±0.018	0.075±0.008	0.030±0.030	0.04±0.12	0.01±0.03	0.22±0.08
1946	9.14±0.05	9.19±0.11	0.84±0.02	0.79±0.02	0.283±0.018	0.076±0.008	0.030±0.030	0.06±0.11	0.02±0.03	0.22±0.08
1947	9.14±0.06	8.58±0.07	0.84±0.02	0.79±0.02	0.282±0.019	0.076±0.009	0.030±0.030	-0.77±0.24	-0.22±0.06	0.43±0.13
1948	9.19±0.07	8.80±0.08	0.85±0.02	0.80±0.02	0.276±0.020	0.071±0.010	0.029±0.029	-0.53±0.18	-0.15±0.05	0.32±0.10
1949	9.22±0.08	9.23±0.12	0.86±0.03	0.80±0.02	0.272±0.021	0.067±0.011	0.028±0.028	0.01±0.07	0.00±0.02	0.21±0.07
1950	9.21±0.08	9.00±0.09	0.86±0.03	0.80±0.02	0.274±0.021	0.068±0.011	0.028±0.028	-0.30±0.11	-0.08±0.03	0.24±0.08
1951	9.16±0.07	8.51±0.06	0.85±0.02	0.79±0.02	0.280±0.020	0.074±0.010	0.029±0.029	-0.90±0.30	-0.25±0.08	0.51±0.17
1952	9.07±0.06	9.11±0.10	0.83±0.02	0.78±0.02	0.290±0.019	0.082±0.009	0.031±0.031	0.06±0.10	0.02±0.03	0.22±0.08
1953	9.05±0.06	9.36±0.13	0.82±0.02	0.77±0.02	0.292±0.019	0.084±0.009	0.031±0.031	0.42±0.24	0.12±0.07	0.29±0.13
1954	9.10±0.07	8.77±0.08	0.83±0.03	0.78±0.02	0.287±0.020	0.080±0.010	0.031±0.031	-0.46±0.11	-0.13±0.03	0.30±0.09
1955	9.11±0.07	8.83±0.08	0.83±0.03	0.78±0.02	0.286±0.020	0.079±0.010	0.030±0.030	-0.38±0.09	-0.11±0.02	0.27±0.08
1956	9.06±0.07	8.78±0.08	0.82±0.03	0.77±0.02	0.291±0.020	0.083±0.010	0.031±0.031	-0.39±0.08	-0.11±0.02	0.28±0.08
1957	9.06±0.07	8.70±0.07	0.82±0.03	0.77±0.02	0.291±0.020	0.083±0.010	0.031±0.031	-0.50±0.13	-0.14±0.03	0.31±0.09
1958	9.08±0.07	8.56±0.07	0.83±0.03	0.78±0.02	0.289±0.020	0.082±0.011	0.031±0.031	-0.70±0.22	-0.20±0.06	0.40±0.12
1959	9.05±0.07	8.43±0.06	0.82±0.03	0.77±0.02	0.292±0.020	0.084±0.010	0.031±0.031	-0.86±0.27	-0.25±0.07	0.49±0.15
1960	9.02±0.07	8.15±0.07	0.81±0.03	0.77±0.02	0.296±0.020	0.087±0.010	0.032±0.032	-1.19±0.38	-0.35±0.11	0.74±0.23
1961	8.98±0.07	8.28±0.06	0.80±0.03	0.76±0.02	0.301±0.020	0.091±0.010	0.033±0.033	-0.96±0.27	-0.29±0.08	0.56±0.15
1962	8.94±0.07	8.54±0.06	0.79±0.03	0.75±0.02	0.305±0.019	0.095±0.010	0.033±0.033	-0.55±0.11	-0.17±0.03	0.34±0.09
1963	8.90±0.07	7.79±0.09	0.78±0.03	0.75±0.02	0.309±0.019	0.098±0.009	0.034±0.034	-1.52±0.46	-0.47±0.14	1.08±0.32

1964	8.84±0.08	8.33±0.06	0.76±0.03	0.73±0.02	0.315±0.019	0.104±0.009	0.035±0.035	-0.70±0.14	-0.22±0.04	0.42±0.09
1965	8.76±0.09	8.61±0.07	0.74±0.03	0.72±0.02	0.323±0.019	0.110±0.009	0.036±0.036	-0.22±0.09	-0.07±0.03	0.26±0.07
1966	8.72±0.10	8.12±0.07	0.73±0.03	0.71±0.03	0.327±0.019	0.113±0.009	0.036±0.036	-0.83±0.15	-0.27±0.05	0.50±0.09
1967	8.69±0.10	8.43±0.06	0.72±0.03	0.71±0.03	0.330±0.019	0.116±0.009	0.037±0.037	-0.35±0.08	-0.12±0.02	0.29±0.06
1968	8.62±0.12	8.67±0.07	0.70±0.04	0.70±0.03	0.337±0.018	0.122±0.009	0.038±0.038	0.07±0.24	0.02±0.08	0.26±0.11
1969	8.60±0.11	7.91±0.08	0.69±0.04	0.69±0.03	0.338±0.018	0.123±0.009	0.038±0.038	-0.95±0.18	-0.32±0.06	0.58±0.10
1970	8.60±0.10	8.19±0.06	0.69±0.03	0.69±0.03	0.338±0.018	0.124±0.008	0.038±0.038	-0.56±0.07	-0.19±0.02	0.37±0.07
1971	8.57±0.10	8.30±0.06	0.68±0.03	0.69±0.03	0.341±0.017	0.126±0.008	0.038±0.038	-0.37±0.08	-0.12±0.02	0.31±0.07
1972	8.57±0.09	8.44±0.06	0.68±0.03	0.69±0.02	0.341±0.017	0.126±0.007	0.038±0.038	-0.18±0.11	-0.06±0.04	0.27±0.08
1973	8.55±0.09	8.84±0.08	0.67±0.03	0.68±0.02	0.343±0.017	0.127±0.007	0.039±0.039	0.40±0.31	0.14±0.11	0.32±0.17
1974	8.47±0.10	8.18±0.06	0.65±0.03	0.67±0.02	0.349±0.016	0.133±0.006	0.039±0.039	-0.40±0.07	-0.14±0.02	0.32±0.07
1975	8.43±0.11	8.08±0.07	0.63±0.03	0.66±0.03	0.352±0.016	0.135±0.006	0.040±0.040	-0.48±0.07	-0.17±0.02	0.35±0.06
1976	8.42±0.10	8.65±0.07	0.63±0.03	0.66±0.02	0.352±0.016	0.136±0.005	0.040±0.040	0.31±0.30	0.11±0.11	0.30±0.15
1977	8.48±0.07	7.76±0.09	0.65±0.03	0.67±0.02	0.349±0.016	0.132±0.005	0.039±0.039	-0.98±0.24	-0.34±0.08	0.62±0.14
1978	8.53±0.05	7.91±0.08	0.67±0.03	0.68±0.02	0.344±0.016	0.129±0.005	0.039±0.039	-0.85±0.22	-0.29±0.07	0.52±0.14
1979	8.47±0.06	8.17±0.06	0.65±0.02	0.67±0.02	0.349±0.015	0.133±0.005	0.039±0.039	-0.42±0.06	-0.15±0.02	0.33±0.08
1980	8.40±0.06	8.07±0.07	0.63±0.02	0.66±0.02	0.354±0.015	0.137±0.004	0.040±0.040	-0.45±0.07	-0.16±0.02	0.34±0.08
1981	8.38±0.06	8.08±0.07	0.62±0.02	0.65±0.02	0.356±0.015	0.138±0.004	0.040±0.040	-0.41±0.05	-0.15±0.02	0.33±0.08
1982	8.30±0.08	7.89±0.08	0.59±0.03	0.64±0.02	0.360±0.015	0.142±0.003	0.041±0.041	-0.56±0.08	-0.20±0.03	0.39±0.08
1983	8.27±0.09	8.22±0.06	0.58±0.03	0.63±0.02	0.361±0.015	0.143±0.003	0.041±0.041	-0.07±0.15	-0.03±0.05	0.27±0.09
1984	8.27±0.09	8.10±0.07	0.58±0.03	0.63±0.02	0.361±0.015	0.143±0.003	0.041±0.041	-0.24±0.09	-0.09±0.03	0.29±0.08
1985	8.26±0.08	7.93±0.08	0.57±0.03	0.63±0.02	0.362±0.015	0.143±0.003	0.042±0.042	-0.45±0.06	-0.16±0.02	0.35±0.08
1986	8.24±0.07	7.66±0.10	0.57±0.02	0.62±0.02	0.363±0.015	0.144±0.002	0.042±0.042	-0.79±0.16	-0.29±0.06	0.50±0.11
1987	8.20±0.08	8.10±0.07	0.55±0.02	0.62±0.02	0.364±0.015	0.145±0.002	0.042±0.042	-0.14±0.10	-0.05±0.04	0.28±0.09
1988	8.19±0.07	7.99±0.07	0.55±0.02	0.62±0.02	0.364±0.015	0.145±0.002	0.042±0.042	-0.28±0.05	-0.10±0.02	0.30±0.08
1989	8.18±0.07	8.49±0.06	0.55±0.02	0.61±0.02	0.365±0.015	0.146±0.001	0.042±0.042	0.43±0.29	0.16±0.11	0.34±0.17
1990	8.13±0.08	8.26±0.06	0.53±0.02	0.61±0.02	0.366±0.015	0.147±0.001	0.043±0.043	0.18±0.21	0.06±0.08	0.29±0.12
1991	8.08±0.09	7.97±0.08	0.51±0.03	0.59±0.02	0.367±0.015	0.147±0.000	0.043±0.043	-0.15±0.11	-0.06±0.04	0.28±0.09
1992	8.07±0.08	8.30±0.06	0.51±0.02	0.59±0.02	0.367±0.015	0.147±0.000	0.043±0.043	0.31±0.26	0.11±0.10	0.31±0.15
1993	8.11±0.06	8.14±0.07	0.52±0.02	0.60±0.02	0.367±0.015	0.147±0.001	0.043±0.043	0.04±0.13	0.01±0.05	0.27±0.10

1994	8.19±0.04	7.77±0.09	0.55±0.02	0.62±0.02	0.365±0.015	0.146±0.002	0.042±0.042	-0.57±0.15	-0.21±0.05	0.39±0.11
1995	8.20±0.04	8.28±0.06	0.55±0.03	0.62±0.02	0.364±0.015	0.145±0.002	0.042±0.042	0.11±0.09	0.04±0.03	0.28±0.10
1996	8.16±0.04	7.74±0.09	0.54±0.03	0.61±0.02	0.365±0.015	0.146±0.001	0.042±0.042	-0.57±0.16	-0.21±0.06	0.39±0.12
1997	8.14±0.05	8.59±0.07	0.53±0.03	0.61±0.02	0.366±0.015	0.147±0.001	0.042±0.042	0.62±0.28	0.23±0.10	0.41±0.17
1998	8.11±0.05	8.14±0.07	0.52±0.02	0.60±0.02	0.366±0.015	0.147±0.001	0.043±0.043	0.04±0.08	0.01±0.03	0.27±0.10
1999	8.09±0.05	7.77±0.09	0.51±0.02	0.60±0.02	0.367±0.015	0.147±0.001	0.043±0.043	-0.45±0.10	-0.16±0.04	0.35±0.10
2000	8.10±0.05	8.42±0.06	0.52±0.02	0.60±0.02	0.367±0.015	0.147±0.001	0.043±0.043	0.43±0.23	0.16±0.08	0.34±0.15
2001	8.10±0.05	7.70±0.10	0.52±0.02	0.60±0.02	0.367±0.015	0.147±0.001	0.043±0.043	-0.56±0.15	-0.20±0.05	0.39±0.12
2002	8.16±0.05	7.89±0.08	0.54±0.03	0.61±0.02	0.365±0.015	0.146±0.002	0.042±0.042	-0.37±0.12	-0.14±0.04	0.32±0.11
2003	8.15±0.06	8.00±0.07	0.53±0.03	0.61±0.02	0.366±0.015	0.146±0.002	0.042±0.042	-0.21±0.06	-0.08±0.02	0.29±0.10
2004	8.11±0.05	8.07±0.07	0.52±0.03	0.60±0.02	0.367±0.015	0.147±0.001	0.043±0.043	-0.05±0.02	-0.02±0.01	0.28±0.10
2005	8.10±0.05	8.42±0.06	0.52±0.03	0.60±0.02	0.367±0.015	0.147±0.001	0.043±0.043	0.44±0.20	0.16±0.07	0.35±0.14
2006	8.04±0.05	8.09±0.07	0.50±0.02	0.59±0.02	0.367±0.015	0.147±0.000	0.043±0.043	0.06±0.08	0.02±0.03	0.28±0.10
2007	8.03±0.05	8.35±0.06	0.49±0.02	0.59±0.02	0.367±0.015	0.147±0.000	0.043±0.043	0.44±0.23	0.16±0.09	0.35±0.15
2008	8.04±0.05	8.27±0.06	0.49±0.02	0.59±0.02	0.367±0.015	0.147±0.000	0.043±0.043	0.32±0.18	0.12±0.07	0.31±0.13
2009	8.08±0.05	8.16±0.07	0.51±0.03	0.60±0.02	0.367±0.015	0.147±0.001	0.043±0.043	0.11±0.08	0.04±0.03	0.28±0.10
2010	8.14±0.06	8.17±0.06	0.53±0.04	0.61±0.02	0.366±0.015	0.147±0.002	0.042±0.042	0.05±0.02	0.02±0.01	0.27±0.10
2011	8.15±0.07	8.22±0.06	0.53±0.04	0.61±0.03	0.366±0.015	0.146±0.002	0.042±0.042	0.10±0.03	0.04±0.01	0.28±0.10
2012	8.15±0.08	8.52±0.06	0.54±0.04	0.61±0.03	0.365±0.015	0.146±0.002	0.042±0.042	0.50±0.17	0.18±0.06	0.37±0.12
2013	8.15±0.08	8.06±0.07	0.54±0.04	0.61±0.03	0.365±0.015	0.146±0.002	0.042±0.042	-0.13±0.07	-0.05±0.03	0.28±0.10
2014	8.15±0.07	8.17±0.06	0.53±0.04	0.61±0.03	0.366±0.015	0.146±0.002	0.042±0.042	0.03±0.01	0.01±0.00	0.27±0.10
2015	8.15±0.07	7.82±0.09	0.53±0.04	0.61±0.03	0.366±0.015	0.146±0.002	0.042±0.042	-0.46±0.18	-0.17±0.07	0.35±0.13
2016	8.18±0.08	7.67±0.10	0.55±0.04	0.61±0.03	0.365±0.015	0.146±0.003	0.042±0.042	-0.70±0.28	-0.25±0.10	0.45±0.18

Table S6. SNP marker information. Primer sequences for the 68 putative neutral (N) and two functional (F) SNP markers used. Sequences can be BLASTed against the Atlantic salmon genome at https://salmobase.org/

SNP_id	N F	Flanking sequence
ESTNV_36086_719	N	ACACAGAGAGCCTGTTTAATCAAGCAGATAGTCCCTGTAAGGATATCAGAAAAGCATCACTGACTCTTTTGTATGCATTGTACTCCAATGTATTTCTTAGCTGAGCTTAACAATAATGGAAACAAAAAAAGAGAGGCGCAAAGCACTGTGCACTTTGGTCAAATCTCACTATTTTCCTTGTGGCACCAAAGGAAAATGCACAACGTTTTACACCAAGAGTACCCCCAACACATCATGGTCCTCGTCTT[C/G]CACAAATTACATGGTGTTGATTTGTATCCTTCTATTCCAAAAGGATTGTGTTAAGTTCAGTTTAGAGACAAGTCCACTGTTACGCAGACGGTGAGAACGTGAGAATCATCCATCCAATAACGAAGTAAAAGAGGAAGACGGGATACACCACCAAAGCTTTGCGATTAGAAGGCTGGCTATCTGCTAAGAAAGCTGTAGAGGCAAACGTGGACCAACTGAATGACGCTGTGACCACAATTAGTCGGACGAT
ESTV_15868_527	N	ACAGACATTGGGGCCTTCCACGAAGTTAAAAGCCCATTTGACGTCTGCTCTGAAGGCACAGGGATTTAAACAGTAAGAGTTGGAGTCCTGCTCTGGGGTGGACAGAATAGTATAGTAGTCCATTTGTTTTAGACCTCATACTCCTAACACATGGGCGGGACGCCCATAACTGTTGGAGACAGCCTTTTTGCAATTGGTATCCTTTAGAACTAAGCTGATCTGGTGAGAGTCAGTCCTACATTAATC[G/T]ATNNNNNNNATAATCTGTTCCCAGAGATGGGGTTTACTGAGACAGCATCTACCGACCTCTGCAAACTAATATAAATGATCCAACCTGACAAATCAAAACTGTCCAGTCCACACACACACACCCTAGTCCCTAGAGTGAAAGTAATTTCTGAGATATCTACATGCAAAAGACTACAAAACCAGGAGGTACAGGATCACGATGATGTCCTAGATTAGAAACGACCCAAGACAATGAGCTTAAATGGGTGAGG
ESTNV_19288_484	N	ACATATTGGCTCTATTTGGGCCCTGGACAGGTGCGTTCATGCAAACCACACAGTATGTGCTGAGCAAGTCCACAACCCACCTAACCCCGGATAGATGGGTACTCTTTCTATGGGACGTCCTTAACGTCTGGTTGATCGGCATGTTCATCTATCTGATACACAATCTCCGTAGAAGGGAAGCCTATACATGGATGTACACCACTCCTGCTGTCCTGCCCTACGGATTCTACTTGTCCTGGATTG[C/T]GAATAGTATTTTGAATATGGCATGGTTATTTCTGTACGACAAAGAATTCTGTGTTTGTCATAGAAAGATGGTGCCAACACTCCTGATCACAGCATTACAGGCCATCTCCAACTGTGTTATTATCATGTTCTCCTGCTACGGACTGGTCGTCTATGGAGCATGGCTCCATAAATATCACAACAATGATCTCTGGCTCATTCGAATACTGGTGCAAAACGGAGTGGCTTTGTATGCAACATG
ESTV_15230_258	N	ACATCTCTTTTTTAAAGATATAAATATGGTCTCCAAATAAAAGTAAAGAAAATACAGCTGAAACACATGGCTGCTTATCCCTTGGTAAGGCCACCCTTTATTGGGCTTCATCAGAGAACATCCAGACATGCAGTCCTATCAAATTGATCTGACTGGGAATTCATAATCATTTTTCAATCATGAGCTTAATGTATGCAATCATGAGCAAATTGAGGATCACACTGTATTTAATCTTTATGGTCTCAAAAGGTTTTGGC[C/G]TACCAAAATATATCAGAGCAACAACAAAAAAATANNNNNNNTAAAAGTGCCCAAAAAAAGTCCTTTGGTTTAGAACTCGTCTCTCATGTTAAAGTGAGGTTGGTCTTCAGGTTTGAAGTCCTCATTGGGGCTACCATCCTGGTTGAGGATGCGTACNGCGGTGTAGCCTACAATAGGGTACTTTGTCTTGTACGTGCCCTCAAATACACTCTCAAGGGATTGGAGCTGCTCTTCAGTAAG
GCR_cBin34264_Ctg1_201	N	ACCACGTTGTCATCCCGTTATTCTGTTCATGTCAGCCATTGGATGTGTTTTAGTCACAGTTGTCCTCTAGTGTGTGAGATTTACAGATGTTACATGTACAAAACGAGCCCTCATCTTTTTCATGTTCCTTCGACCCAGATAGGGTTCATACATTACAGATAGGAAATAC[C/T]TTTTATTAGAGTGTCCTTCAACACACCTTGTAGCCTAGCGGAGCAATGGGCCAGTACCCAAAATGTCGCTGGTCCAAAT
ESTNV_14268_378	N	ACCCTACTCCAAACCTGAACCTGAGACTACATCCCCCACAACCCTCAGCGGCAAGGTCAAATCAGTATCGGGGGCGCCCAAGGTCGTGGAGAAGAAGCTCAAGAAGATGGAGCAGAACAAGACGGCAGCGACACGTTACCGACAGAAGAAGAAGAGCGAACAGGAAGTGCTGAGCGCGGAGTGTTCCGAGCTGGAGCAGAGGAACCGTGAGCTGGCAGAGAAGGCTGACGGCATCAG[C/T]AGAGAGATCCAGTACCTCAGAGATCTGATGGAGGAGGTCCGCTCAGCAAAGAACAGGAAGAAGACCACTGTTAGTGTGGCGTAATCAGCGGAAATTATGGATAGAAGGAAGGCAAGAAAGGGGAATGCTTAGAATGAGATCCAGTACCTTGTGGAGGAGGGGCTACATCCTGGTTATCCACCCTTCTACCAAAGTCTACTTTTGCATACTTGCTGTCTATGATGGGAAGTGTGCATTTGC
ESTNV_28225_649	N	ACTCTCCTAAAGAAGCTCTGGCCCAGTCTGTGTTGGCTGAAGTCCCTGGTCAGGTGGTCACCTACTTCAACATGAATAAGCTGAGTCCTCCCAACACTGACACTCCCCCTGCCCCCGTCCCTACACCGCCAACTACTGCAGACATGCCCACCCTCACCTCCTAGTGCCCAGGAGAGGAACCAACCCCAGTACCCAGCACCAAGCCATCCCCACCTGACCGAAGACAAAGTACACTGTCAGGATGAGTA[G/T]ACAGTCGCTCATCTCAGTAGCTACAATAAGAACTCTCTGCTATTTATCATTGTGTCAAAGGGATCATGACAGGTATACAAAGCTCTAGCCATTACTAAATAGCATGTGTCAGGTTGAATTGAGCAGGATGGAAGTTTTGTAGGAGGAAATTCTACAAAACACTGCATGTGTACTTACAGCAACCAACAACACATTGGTCCAGTCTGGCTTGTTCCCAATCTCCGTCAGTAGGCCANTCCCAGAAGCATGT
GCR_cBin14283_Ctg1_117	N	ACTTTCCTCGTTCTTCCAAGGATCTGTTTCCCACCATCAATTAATCATTCATTGGAATCCTGAGAAACACGGGGAATCACCCAC[A/T]AGGCCTACCTTGCACTGCATGCCTCATTTATGAACCAACTACTTCAACAGGTGGTATTCTGACTGGGTGCCCAGCACAACTGTCCACTCATCTATCTGTTCCGTGTGGGGTCCGTGTTGGTTGATACATTCAGTGTGTGTGTGTGTGTGTGAGAGAGAGAGATACTGTATGTCAGTAAGTGACGCCTTCAGCACCGGGAGCTGTCCATGGTTTTGAATTTCCGAGCAGCAGTGTTCATCC
GCR_cBin13217_Ctg1_178	N	ACAATATGCAACATACTAGAACACATTAAAAAGGCATTCAGCTCTGTCGTAACTGTGCAACAGCTTATAACAAAAACATACAAACATCCCATTTGGCAAACTCATCAAGGCAGTAACAAAACAAAACTCAAACAGGCAGTCCTTTT[C/G]AACTTCTGGCTGTAAGTGCGAGACCTGGGGGCCAGCGCAAGAGCTCCATTTCTCTTCGTAAACCATTTTACTGTAGCAGAACACAGCAGCGGTCTCTCGCATCTGAACAAGGATCTTCTCACTCAGTGGTGAATCCACAGTACACTTTTCTCTTTGAGGACAAAAGTCAAAACCTGACACCACATTAGCGCATTTGCTGTCCAGATACCGGACCAAAAAAGTGCATGTCATTTCCGCGGG
GCR_cBin22215_Ctg1_184	N	AGAGCCCCCACCCCCATTTCCTGGGCAGGACAGGGAGACAGCAGCATACTTACAAGAACAGGGGATAATCCCATTAGCTTCCAAGAGGGGAGTGAGGCTAGGGACTGGCCTGTATTTGTAGCACCAGGACAGATGGTGAATCACATGATCCC[C/G]GGGGGGAGGTATGTGTGGCTATGCGTCTGTGTGTACGTGTGTATGAGGGATGTTAAGGGGACACTGACAGGAACAATAGACGACCAAGCATTCAATGGCTTATTCGACAGGCTTCCATTCTAGCAGTTGAGCCTAACTACCATCCAGAACCATTCACCCTTTCATTTCATTTAGCTAACTAACGTTAACATGAATATTTGATAGCTAACTTATCTACCTAGCAGGGTTCCAGATGCTC
ESTV_17428_1391	N	AGTCAGGCTGCGTCACAATCATTGGTGGAGGCGACACCGCTACATGCTGTGCCAAATGGGGCACAGAGGACAAGGTCAGCCACGTCAGCACAGGAGGCGGAGCCAGCCTGGAGCTTCTGGAGGGTAAAGTGTTGCCCGGTGTGGATGCCCTCAGCAGCGCCTAAGCCCCATCACCGAGGAGAGGGGGGAGTGAAGACAAATTGACCCGCCAGAGTTTGGACTCCCAAGTGAAAAAGCTCCGTCTCCCACC[C/T]TGCATTAGCACCTCTACCATCATCCAGTGACCCAGACGTCAAATCACCTGCTCTCTAGTCTACTACTGTACAGACGGCTGGCACAAATGGGAATGCGCTCTCCCTCACTCCTGCACCTGCCCCATATCTTCATCACTGAGGAAGTGGAGGCGTTCGTAGCTGAAGCCACCCTCACATGCACTTATGTTCCTTCCTGCTGTGTTTCTCTAGGAGACGAGGGCCAGAGACCAGTCAGCTGCAGCTAGTTGTT

ESTNV_22611_642	N	AGAAACCAAAATAGGAAGCCACTTTAGACTATTGGGATGCAGCCTTCGTCACAATGTGCTGGACGTCAGATTTCTGATGTAATCTCCATGTTAACGGAGACAGGCTTGTGGTTAGTTGCTGTCACACTGCAGTAAAACCTTTGTGCGTTAATTGGGGAGTACTATAGTATGAAGTTGCACCTGGACACTGATCTATGGTCAGTTTTACATTTGCACCAATAAATGTTAAGGCTATGATGTG[G/T]GTGGGGTAGACTGATCCTAGGTCTGTGGCTGACAACTTCTACCCAGAACCAAGGGGATGGCACTGTTAGGAGAGGCTGGGACTGTGTACATAGACAAATATCAGATGAACATTTTTATATAATTACAGAAAAACTTTGTCACCTGTTGTCACACCTGCTGATGTAAATATAGACTTTATTTTGTTTAAGAGCACTGTGTAATCTAAAGGTGTACATTTTCCAAGTTGTGGTTTGTTTTTAGAAAATAAGT
BASS111_B7_B11_707	N	AGAAATCCCTCTTTTGAAAACCAGTCCTGTATAGCTAATCCTTCTTTGGAAACAGACTCCTTGCNTGTGAATGTGCAGCGTCATAAGGAAAGGCTTGTTCCTTTAGCTGACTGCTTGCACGCCATNCCTTTCAGTACCCATGCCCTATAAAGCTACAGTCACAGGCCTGGACAGAAAACCCTGAAAATAGACAACAACAAAGTGAGGTTAAATGAGGGATCCACTAAAAAAAAGAANGGTAAATCT[C/T]GAAGACAGATACCTCTGTTAGAAGATGGAGGGCATTCAGTGAAAGCACAAGCTAGCCAGCTCTTTTCATTGCTCTATTTCCTCAACTGTTTTCTTCACAGTTGGAAGCCAGTTGTATGCATGCAAACTGCTCTTAATGAGAGAAAGAGAGATTACTCAGGACTTGGAAACCCCAGATCCCGCAGAAGAGTTAACATAATATATGGTATTGCTTAACAAGAGTCCCACCTCTTTCTCCATA
ESTNV_33766_718	N	ATGATGGACAGGTGTTACTTAGTTGTGCGTTTCATTGGTTCGTTTGTTTTTTATCTATGTTTTTGTTTTCTTCTCCTGTCTTTTATTTTGAAGGTGAAGGAGCAACTTGCGCTGACCTTTTTGGTGCCTCGTGTGAGGATAGCGAAGTTCCTCATGTTCACTTTTTCAAATCCTTTCTCCCTTTAACCGTTGACATTCCTGATCCAGTCACACCATTCCTCTCTTCCTCTTTATCCC[A/C]AAAACAATCCTTGAGTCCCTATGGCGTGGCAGCTTTACCAGATCTGATTATCCCCCTTATTACCTACAGACGAAACTCTCCATCTTTGAACCTGAGCTTCTCCAGAAGGGGTTCGTCAAATGGAGGTTGCACTTGTCTTTGTAGGATGTCGTTCAGGGATTTACCNNNNN
ESTNV_36692_1354	N	ATGCTACACGTCCTGGAGGAGTGGTGGTTCTAGTGGGGTTGGGTGCAGCGATGACCACTATCCCACTGCTTAATGCTGCTCTCAGAGAGGTGGACATCAGAGGGGTCTTCCGCTACTGCAACACCTGGCCAATGGCTATAGCGATGCTGGCCTCTAAGAAGGTGAACGTGGCGCCCCTGGTGACCCACCGGTTCCCCCTGGAGCAGGCTGTGCAGGCCTTCGAGACCACACGC[A/C]AAGGACAAGGGGTCAAGATCATGCTCAAGTGTGACAAGACTGACCAGAACCCCTGAGAGGACTGGGAACACCAGGACCAATAAGAGAGCCAGTCTAGACAACCACAGCAGCTTTCTCTTTCATCATGTGATTTCAAGTTGCTTTTGAGTGGGATTGCTTGATTACCATATTTGTATAAGATGACGCAACACTGTTAAATAGAGCCTTTGAATCTTATTGTAGTTGTTTTTTTGTGTAGTC
ESTNV_23996_351	N	ATTATACTTTTAATAAGAGGCATGGAATATCACCGGCCTACATTTATGAGGCTGCACTCCTGAAGTCAAAAGATGGCAATAATATAATGTCAATAAATGACCTGATAAATAAATATACATACAGTACTTCAGAAAGGACACATTATTTCATATAAATAAAATCAAGTATCCTAAAATAAATCCATGAGAAAAACTAAAACAATCAAATTAAGCAAGAAATACAAGGGAAAGATAAAAGTACAGTAACAGT[G/T]TTGTTTATGTACAAGACAGAAGGTCAGAAGAATGCACACCGGAGGGAGACAGTGGGGAGTTACACATCTCTACTGTTTGTGCTGTACCGCCATCTCTGTAACAGCCTGGTCCCAGATCTGCTTGTGCTACCATGCCAACTCCTTGTCACTCACCGTCATGCCAAGCATGTTTGACATGACATTGAGTGGCAAGGTGTTGACATGATGGCCCAAACAAGACTGGCACTCAGGCTAATTTGCTAACACTTCC
MHC_IA_33360-33530	N	CACCTGGTAGTGATCACGGAAGAGGGGGTGGAGCATGTTGTCATCCCTGGAGACAAGCTGCCCAAGTTCCA[C/T]GACGAGTAGTGACACTTCAATCAGCCAATCAGCTGTGACACTCTGTGAGAGGAATTTGGAACCTTCACCCAACCATTCACTGTTGTTCATACTGTACATGCAGCATTT
GCR_cBin5337_Ctg1_532	N	CACTGGCTGCTGACACTGGAGGTGCGGCTGCCGAAGGGGTGCAGTGGGATAGTGCCGATGACCAGTGGCAGGTTCAGAGACAGGTTCATAGCTCCGGGGATGTCCACGTACACCTGGAGAGGGTAAGCAGCACACAACTGAGGGTTAGAACTGAAGGCCCACCTTCAATGAACAACAAACCTGTGTGTT[C/T]AACAGTGTTAAGACTTAGGCAAGGGGCAGTCCCGATGACAGGGTAAAGAGATCATTAGCACCACTAAGACAGGTGTTGCCACTAATAT
ESTNV_35893_841	N	CATCTTTGACAAATTAGAAAATGCTATTGTGTTTGGATAAGACACTGGCTGGTGAGCAGACTCATCTGTAGGGTACAATTGTCCTAGACCAAAACATCAAATTGGTTTTGAAACTATTTTCTCCCTGTAGTTACTTTGTTTGTCCTTTGGGTCTTTAACACTGTGCTCTATCAGTGTTCT[C/T]CTGATCCCTTTTTGTTTTTCTCAGTTGAACAAGCCTATTTAAAGCAATAGCATAAGAGTTCATACCCTTTAACTCCTAAAAGATGTCTAAGGTGGCGTGTGTGTGCTACCATATGGCTATTGAAATAGTTTTTATTTAATTGTTCTATTTTATTGGGATGTTCATTTCATGGAATTGTAAGTTTGTGTTTGAGGGATTTGAACCAGTGTTTATCTGTATCTAGACACACAATCCATAGAC
ESTNV_35759_1059	N	CCACTACCCAAAGCCACAGTGAGGTTTCACAGGGCAGGATCGATGAGTCAGCCAGATGACGTGTATTCATATTCACAAATATTCATGGGAAAGTGGGTTGTTGACATTGAGAAATCGCCTAGTCTGAGATAATGAGACAGCTGTAGAGGTAGTTGGAGGAGAAATTCTTTGTTTCTGATGTGGGAAGAGTAATGTGGCAAGAATGCTCCATGGAAGGGTACAAAATATTGTGGACAAAAAAACAAAAA[A/G]AAAGACAATCATCACAAATGAGCTAGAGCTGAGGATTGGATAGTAAGAACTGGCAGCCAAGAACCTATTGCCTTTTTCGACTATCTTTTTGTTGTTCTGGAAATATAAAATAATTCTCAATGTGATATCTGGCTAACTGTTTCCTCCTGCTGTGTGAGATCCCACCTAGAACATGTATATAATGTTTCTACTCATACGGAACCTTTTTGTAAAGACTAAGATCATTGTACAATGCTGAAG
GCR_hBin32129_Ctg1_119	N	CCCACAGCCCAACACTAAGAAAGGTGAGTTAAAGGGAAGGGACATGAAGTGTGATATTTGTCTGCCAAAATGTCATTTTCAAAGAAA[G/T]AAAAAGTTGAAGTTGATCATGGCTGAATCTGTGTGTTTTCTGCTTCTGCACTCAGCCTCTACAGACGAGTATGACTATGCCACATCTGAGAGAAGCCCCACGTATGAATATGAGGAGAAAGTGAGAAACATCTATATTGTTTTTATTGCAA
ESTV_17429_1139	N	CCCGGTAAAAATGTAAGAGAGGAGGGGTTGGACATGTTGATTTCAGCGACTCCAGCCTTGATACTGCATCCAAGAAAGATTGATCCAAAAGTGCTCATGATGCCTGGACACCAGATGGGATTACTCATTCAATGTGATAGTCTACTTTGTTGCAGTAACTGAACTCTTCAACCAATCTCTTTTAATACAATTGTTTTGGCTAAAGAATTGGTTGTTTGTACTTTTGTTTCCTTTTCCATATTTNCCTA[A/T]ATTGGGATTGAAATGTTTTACTCTACATCAGTTGGGTAATTCACATGAAACCAGNNNNNNNNNNNNTAAAACATGTCTGTCGCAAATTGAGTTACAAAACCATGATGCGTTTGCAAAAATCAACTATCATTATGAGGACTACGGTCAACATTTTTGGCAAAGTGTCTTATTTTAAATTGTACGTTCTCGCCTGAATTTTGTACATTTTCAGTTTTCATTACCTTTTTCGGATTAAATTATCAGTTATTTAA
ESTNV_28129_298	N	CGCAGCCATATTTAAATACAAGTATTATCTATTACAACAGTTAGGTTAACGCAGCACAGCCAATATGTTCAAGAGGGGAAATCTATATGGNGCTAACAAGGGTACAAGCAAAAAACAAAAAAGCCATCGATACTTCAGCAACGACACACCTGACATGGTTAACTGACGGTGTAACATGGAATGCAGCAGAGACGACTTATTTCTTGGGACTTATTAAATAAAGGACAGGGAAAATTGAAAAAAATTC[A/G]ATACTAAAAATGCCATACCGTCATCTGTGGAGAGACCACTGACTCAAACAAACCATCAGTACATACAGAGGTGTTGAAGTGAAGACACTTATAATTAGTGACACAGAGGAAGCAAAAAAAAAAAAACTCCCATCTTCCTCTAGTGACTATAATTGGTACTGTGATCCACTAGACAGTCACATAATTCCAACGCTGTGCCAGGAAAGGAAATGGACTTGACAGTCCGAGGTGACATGATTCCTCAAAATTA
GCR_cBin21600_Ctg1_99	N	CGTTGAAGTGACGTCTTTGCCCAGTGTTTTTTTGTTGTTTAGTTATTCCAGTTAGCAATAATCTAATTATTGATTCACAGCC[C/G]CTTGTGAGTGGATCAAACTATTTTTAATGGTTGGTCGCTAGATTTGTTTCGTACGTGCCAATTTTAAAATGTGAAAGTTATGTTTAGTCACTGCAAAGAGAGTTTGACTGTGAATAACCAGCTAACAAT

GCR_cBin15343_Ctg1_36	N	CTAGACCAAATCTAGGAATACTTTCTCTTACCAAA[A/G]GTTTTACTACAGTTTTAGTCATCACAGTGCATAGTTTGAAATGCTTCAACCCACTTGGTAAAGCGGTCAATTATTACCAGCAGTATCTCCTTTTTTTCTCTTTCGTTCAGCCATGTCTATGAAATCCATAGCTAACTGGGTGAACGGGCCGGCCGGCGCTGGAAATTTCCCTGGTTTCAGGGGGGTACCCTTACCAATGTTGCATTTCATGCATGTAGTACAGGTGTCAGATATTTGTCTTTCCATCACC
GCR_cBin48270_Ctg1_181	N	CTAGAGTAAATCATGAACCTAGGCTGCATTGCTAAATACCTCAGCTACTTCGGACTAAATACCTCAGCTACTTCGGACTGTCCACAAGTGAAAGAGAAGGTGAAGCCCAAAGCTTTCGTCTTCTGATTAATGTGCTTCTTTTGCAGGAAGTATTTCAGAGACTCACATGGTCAAACAGCT[A/G]TAAAAGAAACATGGTTGGGCAGATTACTTAAAAAGAAAAAAGGAATCGATGACTGATTACAGATTACATGACAATAAAGGTAATTAGTAATGTAATCTGTTAACTCAAAATGAGTAACGCAATCTGATTCCTTTTGCATTACATTTCCTAGGTTAGACAGAATCGGCCTCCTCCGTTTATGCTGCAGACCTATACGTGTTGTCTCTAACTCTGCCCACAGAACTGCCCAGCATCCTTGGGAAAGTACTGG
GCR_cBin40148_Ctg1_35	N	CTAGATCTTCCTCAGGCCAACAGACATGAGGGTG[G/T]TTTATTCATCATCAGCTACTGAGATACAGTATACCGGGATACACACTGAGGCAGGCATCAGATAACACAGTGATAATGACACAATGAGTCTGAACTCTCGCCTCCGATCCCCACAAAAGTGTTTCTTTGTACAGGATAGTGTAAACACAGGCGTCAGTGCGTGGTCGTATTGACAGCGCAGTGTAAAAAGTTAGCCGTGATTTCGGTGTTGAAACAGATTAGT
GCR_cBin17883_Ctg1_169	N	CTAGATGACCGTCATTAGTGGTGGCCATATTAGCAGCACATACTGTCTATATGCTACATTACCCTGCCTCTGGCTAACCTAAGTGAAAATGAATGAATGAGTGAGTGAAGGAATACGCACAGACAGCACTGCCCACCCACTCCTACAACTTAAAGCCAAACCCTGACG[A/G]CCCAGCCAGAAGTCTACATCGCCATATGGGGTTGTATGGGAGAGACAATGGCGCAGAAGGAGATGG
GCR_cBin49912_Ctg1_98	N	CTAGATGTATATACTTGTGTGACACTCATTGTACACCATGTGCGAGTTACATTTCTTCACATTCCTTCATTCTATTAAGCATGAATCATTTTTACAT[G/T]ACAAAATTGCTGTCAGAGTGCCGTCATAACGAAATTGATAGTAAATTATCCAGTCGTCATCTTTGACAGCCAATTAATTTCGTGCTTGTGAAAACAGTGAATATACCTTATCCAACCGCAGCCCAAGAAGAGGACAGAGACCCTCCTTTCTGCCAATGCGCAACCAACCAACCGCGGGCAGTCATTAATAATTCAGATAGTATTGCTTTGGGGAGTGACGCGGAGGATTCAAATGCTTGA
GCR_cBin32565_Ctg1_59	N	CTAGAAATACTGTTATCAAGACCACACTATATGTAGTGGGTTACTGTGCAATTTGTTG[C/T]GAGGATTGTGCAATCAGAGTGAATGTGGGCTCTACATGAGGAGTTTAATAAACTAAACAGTCAGAGTGAATGTGGGCTCTACATGAGGAGTTTAATAAACTAAACAGTCAGAGTGAATGTGGGCTCTACATGAGGAGTTTAATAAACTAAACAGTCAGAGTAG
GCR_cBin5182_Ctg1_123	N	CTAGAAAAAGGCACTTAGATGGTACATCGGACCAAGCTTAGCAGAAACAAAAGTGCAAGAATATATGTTGTATTGCGTTCTTGTGGTCCAATGATTTCCCCTATTTCTTTCCATTAAATGAT[A/T]GCATTTTCATGTTGCTGGGTCTTGTAGGCAACTTGGGGAATGTCTACACTTGACACTTACAGTACATGCAAATTCTGCTATTAGAATAAAATGTTTGCATTGAAAAGACATGTCTTGACACACAGAAGTCGCATTGCGGTCTGATTATGTTCAGATTTGGTCAGTGACACATCGTATGTAGATTTCTCCTCAG
GCR_cBin15671_Ctg1_125	N	CTATGATGCTGAGTGGTAATATTTCTCCCAACACTCTCACTCAACTTTCAACCCAGAAGTGCTATCTATCTCGGCTTTTTAAAATGTGACCTA[A/T]ACTAATGGGCTATATGTGGTGTAAGGGATTCTCTTTGTACAACAACAACAAAAAAATAACATTTCAAAGATGTTTCCTAGTGGAGGGAATCACTAGGCATCAGAAAGAGAAGTGGGATTTTTTGTTTCACTAGACATGCATGAAAGGTCAAAAGTCAAAAGAAAACTTGCCGGTCATCCTTTGCTCTCCACGTTTCTATCACGTTGATCGGCTCACGCCTCAAGATTATGCCTACTCTTC
GCR_cBin3299_Ctg1_307	N	CTGTATTTCTATATCAATAGTGCTACATCTGAACTGACGCAGTGACCTGTCCACCACAGTAGCCTTGGTGGATGACAGTTATTGCTATATCTCCTAGTATGGAGAGACAGGCAAACCTTGGATGAAATCATAAGAGCACCACCAGTGAATGGAACTGGCCTTCATAACATCACACCATCCCAGCACCAGACTAAAGACATCCAGGTATCTGGGAAGATAGTGAAGGTGGCAGC[A/T]GTGGTTTCCATGGTCCTTTTCTGATCAAGGGGGACCGTTGCCCATCACTTCCAAGCGATTACCTCTATACAGTAAGGGATTAGTATTGGGATAGAACAAGACCACGCCAGCAGGCAATCTGTTTTTCAGTTGAAAACTCCCTTGAGGTTTTCCATTATCCCCCTTCATATCCAGCCTGACCTGGAGAGAAAACCTGGCCTTGGAGGGTCAACTCAG
ESTV_14201_395	N	CAACGGCAACAGGACCGTACCACCATTCCCCGAGGGACTGCAGATGGCTACGTTTGGGATGGGCTGTTTCTGGGGCGCAGAGAGAAAGTTCTGGAAGCAGAAGGGAGTCTACTCCACCCAGGTGGGCTATGCTGGAGGCCTCACCCCCAACCCCACCTACGAAGAGACCTGCACAGGTAGGACAGGCCATACTGAGGTGGTGCGGGTGGTCTGGGAGCCAGAGAAGACAAGCTT[C/G]TCCAAACTGCTCAAGGTGTTCTGGGAGAGCCACAACCCAACTCAAGGAATGCGTCAGGGCAACGACGTAGGAACGACCTATCGATCAGCCATCTACACATACACACAGGAGCAGCTCGAACAGGCTCTGGCCTCCAAAGATGACTATCAGAAGGTCCTGATAGAGGGGGGGCTTTGGAGAAATCACCACAGAGATCGCCGAGGCCCAGGAGTTCTACTATGCTGAGGACTACCACCAGCAGTACCTCAGCA
GCR_cBin4844_Ctg1_156	N	CAAGGTCATCCACACTGAGTACCTTCTCTGCCATCCCTACCTATACCAGATACTGTGTGTCCCCATTATTGGGTGACTGACTGACAACAGGGCGGATGATCCTCTTAACTCATCCCGCTTCGTC[A/C]TTTGTCATTTGTCAATAACTCAAAGGTGATCATTTACTCCATGACGAATGGCACACGCATAACACCACTACCTCTGTTAAATAGAACGGTACAGAAAAAATCCATTACAAGCAATTGCTTTCCTTGTGCAGTGGCTGAATGTGATGTTCATCATCACTGTCAGCTGGATGGGATGAGGACCAAAGGGCTTTCGGAGGGGAAAGCTCATGCTCCATCTGTTTGTGTCCCAAATGACACCCTATCCCCTATG
ESTNV_32013_264	N	CAAAACATCTAACTTTATTGTTTCATGTGATAAAAGACAATTATTATACTTTTTATTGGCTATAATGAGTAGAGTATTTGCTCTTACAGATATGTTTGAGTGTATTAGCTTTTACAGTACCACAATAACAGGATGCACTACAAAACTTCCTTTAGTGAAAGGAGCTCGTCTTAACATTAGACTGATTAAAAAACCAACAAACTTTTTAAGAACAGTCAACCCATTACTTTACGATACT[A/G]CATATGAACAAATCCCCTGCCTAGTGTTTACTGAGAGTTAGTGGTTGTGTTTGAATGCTCAGAAAGAGATGTCTTTAACAAGCTTGTATTGGTGCTCAGTGTCTGCGCTGGCACATTTTACGAATGACATGGCGAGCGTTCGCGTCTGGCTTAGCAAATCTTTATCACTCTGTTCCAGCTGTTTCATGGTTGCTGTGATTTGATTGGTCTTCTCCTCCAAACGAGTGATCATGTCGTTCTTCTGTTTCAT
ESTV_19099_538	N	GACCCCAGACCAAGGCAAAGCCCGGCTCCTGACACNTTGGGAACAGTTGGACTACGGCGTGCAGTTCACATCATCCAGAAAATTCTTCACCATCTCCCCAATCATTTTATATTTTCTTGCAAGTTTCTACACGAAGTACGACACAACACACTTTGTCATAAACACTGCCTCCCTTTTGAGTGTGCTGATCCCCAAATTGCCACAACTACACGGAGTCCGCATCTTTGGCATCAACAA[A/G]TATTAAGCTGAGTGTTGAGGGTCAGTGTTGGGACAAACTCTCAAAGGAGCTATTGGCTCATAGCAAGATGAATAGTTAACCCTATTTACTCTGTACCACTGGAGTTTGTACTGAGCAGTTTTGTACAGTATGTGTAATTGTCACGCAATGTTTAGAGACGCCAGGTTAAACTATAGTGGGAGACTTAAAGAGCGATAATTCCATGTGCGTTACTAACTTA
ESTNV_31647_643	N	GATTGTTCGCGGAGACTATTCACAGCTGCCTGAGATATTATAGAAAGGAGATAGGAATCCCATTGGGCAATGAATTGAGAAATGCTGCAGCCAGCGGTTGCTAAACTAATCGTCACGCAATCCCTTCTCAAAGTCAGGGTATGTTGGTGCTTTCAAGACAACTGGGAACTCGGTCAAATCAAAACATCAGTGATTTTTCAGGTCGGAAATTCGGTGATCAAGAAAGATGCCTGTGTTTCCGA[A/C]TTGGAATTCCAGTTGGGTGACCATTCAAAACCATTTTTCACTGTCGGAGCTTATTTTTTTCCCTAGTTCCAAGTTGTCTTGAAGTCTGAGATTTCTGAGTTCCCAGTTGTNTTGAATGCGGCATGAGTTGAGAAACCGGCCTTTTTTCCTAGAAAGTATGTAATGGCACGATTCCAAAGCTATAAGATATTACAGAGAAN

BASS119_B7_E10_373	N	GCACGCGCAAGACTTTCATGAGAAGAGAACCCAATCAAAACCCTAGGAGAGTCAAAAACTCTGACAAACACAAGAGGCTAATTAAAATGTTACTTAACCGCACTTTAAAAAGCACAACAGGAAAGGAAACTCC<br>ACACCCTATAAGAGCTGCGGAGGAGGAACCACTATAGCCAGCCAGCCCATCCACATTGAAAATGACACTACACAGTGGTTAAACACAAACAGATTGTACATGTCCACAACAGAATGATA[A/C]ACAAGCATCA<br>AAACAGGAGGGTGAATCAGTGGTGAACACATAACTAGAAGGAAAGTATATGTTTGTGTGTCATAGTTATATTTCTGAGAATGAGGGGTAAAGGTAGCGGAATATGACAAATATATATTTAACGGTATGGCAG<br>TATTTCATTGTTTTTGAATAATAAAAAAAAGTTTGACATTTGCTTTGAGTAGTGATTGACCCTAGGGAGGCAACAAANAAATTGTAAGCGAATAAACA
ESTNV_23346_206	N	GCAGAGGTTTTTCTTTTTTTTTTCTCTCAGTAGTTTTTTGTTTCCTGGTAAATCTTGGTGTGCTTTCTGCTTGGCACAATAGCACAGCCCACTTAACTTAAACTCATAAATATGGCTGTGATAAAATAGTCACAAAA<br>CAGATTTTTTTGGTTCCAAGATTTGTGCAGGGAAAAGATAGAATAGTAATCATAATGTTCAGGTCATC[A/T]AAACATCAAAAATCTTCCTGTCGGCAAATAATGCCCTTTCTTTGACATCCCTCCTTCTAAAAAC<br>ACGCACCGAGCAATATAGTTACAGATTTGACAGAAAGTACAGTCAAAAACTGAAACTGAACCTCATTGATAGCAGTTTACAACAAAACAGGAAAAAAAATTATATAATTGAACAAGACCATAATAACTTCTTTC<br>ATAAATTAAATAATATTTCTTAAGATGATTGGTCTATTGCAA
ESTNV_36576_1908	N	GCATGGTGGCCATCAGAATGTCGTGACCGGGACAGTCCACGAAGGACACATGTCTGACCAGTTTGAAGTTGCCCTTGGTTCCAGGGATGTCTGTAGGGAACTCATCAGGAGTACTGGAGCCACACGACCTGT<br>AGCACTCTGGCCTGGGACAGCTGGGGTCGTCCAGCTTATACACCTTGGCGTTAGCGTAACCTAGCTTGATGGTGATGTTCCTCTCCAGCTCGTTCTTGAAGCGGACAGTGTGAAC[A/C]CCTGAGATGGCCTTG<br>ACCACCGTTGACTTTCCGTGGGCCACATGACCAATGGTGCCAATGTTGATGGTGGCCTGTCTGCTGATGATCTCTTGGGAGAGAGGGGTCAGGATGGACACATCCAGCGAATTTAAATCTTGTTTGGAAAGAT<br>GAGGCTGACCCAGCGTTATTCCAGACTCGTCACCCGCCATC
GCR_cBin11008_Ctg1_366	N	GCCAGGGCCAGGCTCTGGAGGTCAGAGGAGGAAGAGGAGTACTGAGACAGGAGCTCAGTGGAGTCACACGGGTCACAGCTCACTGAGAGAGAGGATAGAGGAAGTAGATAGAAGGGATGAAAAACTAGT<br>AAAATAGGATTTTAGTTGACCATTTTAAAAACAATAAACAAAACATATGTATAAATCAAATCATTGAGGAGGACACAGAAAACGGTCAGAAAACCGTCTCCATT[G/T]CAATGAATGATTGTTGGATGGAAGAG<br>GAATCAACAAGTTGAGAGATATTTGAGTAATATTAAGCTCACCAATA
ESTV_17112_405	N	GCCAAGCTTGGATGTGTGTAAACACAATTATTTTTTGATTGAATGGTCTCACAACGCTGTCACATAACATCTACCGTCCCCTACATTCTATTAACCCCTGCCCTTCTCTCGCCATACATGAACCATCTCCTCTCTATG<br>CCTTCTCTGCCTGGTACTGTTGGTGCAGCATTAGGACATCTGACTGGGACACTCTGGTCCAG<br>CCCTCACTGTGGACGTGGTAGAGGTTGACCTGTCCTCCACTGTA[A/G]GCGTCACGGTATGTGGCCTGATAGATGGCCCTGCGACCCAGATCACAGGCTTCCTCCACTGTCAGGTCCTTCTGCTTCAGACCGCTG<br>TCCACGATGCCATACGCGTACATGGAGCCAGAGCCCACAGCAAACAGGTCCCCACACACACGGTTCCCCTCAGAGTCCACGTAGTAGAGACCTGGTCCAGTCTTGTCCCAGCCACACACCATGGTTCCCATGCT<br>AAGGCCCATGCCTTTGTACTGGTACACCA
ESTNV_31902_575	N	GCTCTCTATTGGTGAAGAGAGGTACTGAAGCCAGGCAGAAATGGAGTACTGACTGTCCTAGGAAAGGTGAGAGTTCACAGATCCTCATATAGGTCCACAGACATGAGAGAGCGCACTCATTTCAAAGGCTTAT<br>ACCCCTAGTTAGTATCACGGGCACACAAATATATCAGAGTTCGAAAAGAAAACGTTTTTTACCTCAAGAACAAGACTTAAGACACTGGTGAGAGTATGAAA[A/C]AGTCCGTCCCTTCGGGTTGAATCTTAACTT<br>GATAACTGTACACATAACTCACAGCTTAAAATGTTTTTCTCCCATTGACAGCAGTGATTAATGTGCTTAGATCTCAGGTTAGGACTCAGCCGTGAGGATCCATACCACTAGCCTGGAATCCAAACTGATATGATC<br>CATTTCACCATTGTGTCACAGAGCATACGAGTTTGGTTTCCAGGCTACCATATTGCAGGCATAGCCATCAGGCTC
ESTNV_34021_1870	N	GCTGTCGTTTCGTGAATAGCAGTCTGGTGTCTTGCAGCAGAATACAAAAAGAGTTCGGTGAAAACTTCTATCCTGTCCAGTTATTGGAGCGTAAACTTGCAAGAGAAATGCGGTAGGAAGTTGGCATTTCCCAC<br>ATGCTAACTCAGGAAGACCTGGGATGTCCAACTGACTCAACCATGCAGGTTTGCCTCCAACTTTGCTGGGGAACTGATGACTCTGTAGTCGCCAGTGTTCGGCCTCCTCCAAAAA[A/G]CCAAGAGCAACCCCT<br>GTTTCTGCCATAGTGTTAATTTTACTATTTACCAAGTAACGTTGATTTGATCGTATTCCTTCAATCGCTGGCTAGCTGTCTGTCACAACAAATTCCAACGTGCGTCACTG
ESTV_17611_91	N	GCTTGGAGTCGTGATCATCCTCTCTGAGTTGTGTAAGTTTGAGTGTTAACCTCAGTCTCCCTCCACAAGAAGATGCAGGACTTCGCCTTC[A/G]CTGCCCTCTCCATTTGGCTGTGTCTGGGTGCCACCGCCCTGG<br>CAGAGTCCCACGGCCCACAACACTGCACATCACCTAATATGACTGGGGTCCTGACTGTGATGGCCCTTACAGGAGGTGAAATCAAGGCAACTGGACATTACAGCTACGACTCTACGGACAAGAAGCTACGTTT<br>CACTGAAAGTGAGATGCACCTCAACAAGACTGAGCATCTGGAGGACTACCTGATGCTGTTTGAAGAGGGGGTCTT
ESTNV_35257_1292	N	GGGTGACCACCGATACAAAAGTAGGAGTACACTGATACAAAAGGAGAAGTAGGTTACCACCATGTCTCCCCTAAAAATGTTTTGTTTTTCAGTTTAATGGGGGAGAATTTGACAAAGCCCCCCAAACTACAACT<br>TCTGGTACTCTACAGTTGCTAACGTGGTCACCTACCTCCATCACTAACCCATTTCCCTTACAGAACTGCATTATGGATAATGCAGTTCTTTAAGGAGAAAACCTGTAACAAACTTA[A/T]TTCCCCCTTTCTCCTGG<br>TTTTTGTTCTCATGGTACTTTTTGAATAAGCTCACTATTTATTAGCCTACATCTGCTTCATTGACTAGGTGNNNNNNNNNNNNNNNNNNNNNNNNNNNNNNNNNNNNNNNNNNNNNNNNNNNNNNNNN<br>NNNNNNNNNNNNNNNNNNNNNNNNNNNNNNNNNNNNNNNNNNNNNNCTAAGGGAGTAATGCCCGGTCAAGTGGGTTTATCCGCTGCCACCTAGCGAAGGTTAAATCCT
GCR_cBin47268_Ctg1_70	N	GGGTTCTCGTATGGGGACAGCCCAAAAAGCCTTTTTCTAAGAG[C/T]GTATCAGAGGTCAGCTAGGTCACAGCTAATTTTGCCACAGGGTTCGTTTCCTACAACGCTCTCCACTGAGTCACATTATCCTCGTTATC<br>TTTTTCATTGTTGTTGTCGTTTGGAGTGTCCCAATTTCTCTTTTTTCCCCTTCTGTTATATGCCAGCAGGGATTCCTTTTTTCCTCTCCTTTGGTGTGGCTGCCGTTCTTAGGATGTTGGCTTATTGAATTTAGT
GCR_cBin2854_Ctg1_228	N	GGTCATGTAATGAGTACTGTATTGTAAGGTCAGGGGAGGCATGATGGTAGAGATATCTCTGCTATGCACATAGTATCTCTGTGGTGCACTGTGCACACATCCTCACATTCAAACATACACGCACATCCAAACAT<br>TCTGATTATGAAAATAACCTTTTTCTAAACAGTTCTGTTGGCTCTACACGACAGGATTAGAGTGAGCCTAATGAGTCAAAACCGAATCAAATC[G/T]TTCAGGTCACTACAATGAGACCTTCAAATTATTATTTTT<br>TCATTCAACTCAAACATCCGAGTGGTGTGTATAGACATAATAAACGAGCAGTATCAGTGACACTAGTAGAATCACCAATAAAACCAACATAGCTTACCGCAATGTGTGGTGGTTTCATTGATCAGGAAG
GCR_rBin4036_Ctg1_280	N	GGTAAGGTGTCTAAGGCCTGGCAGAGCAGCAAACTTAGGGGGCACTAGTGCAGGGAACTGTGGACTTAGAATGGCAGAGGACTAGCACTAAGGCAGAAGTCCAGTAACCTAAGTCAGAGCAGTGTGAACCT<br>GCAACTAGGGCCAGAGACTGAAGGCCGTGTATGGACAGAAAGCCTACCGGGGGTTAGGGTATGTCTGACCTACCATAGCGGCAGGATACTTATGACCTACTGATGAGG[A/C]AGGATAGACTGGACCTACCA<br>GGAAGGC
GCR_cBin23727_Ctg1_154	N	GGAACACCATGCTCTATGACTCACAAAACTAAAAGGCCACCTAAGTACAAGCTGGCTATTTACAAAGACAAGTAGGCTATAACATGGAGGAGATGATGCCAAGGTCAATGTTGTCCATAATGAAAA[C/G]CAG<br>AAGGTTAAATACAGATGGAGAATTCATACAGAATGAATTCTGTGACAATTAAAATGTAGGTACACTAGAATGATGTAGGCTTCATTGTAATGAGGCCTTTTCACTATTATAA

GCR_cBin17226_Ctg1_170	N	GAAAAAAGTACCCTGGCTGGCATAATGAGGCAGTCTTGTGTAAGGGCTCTCCTCACTCCTCTGCATCCTTGATGAACTATTGAATATGACATCAAACGAGCCGAGGGCTGTATTTTGAATATGTAAGCTACTGTTGCT[C/T]TCCAGCCTGGTTTGCACATAGTACAGTAAATCAGCTATTTGAGCAGATAAAATAATTTAAACATTTCTTCTTCCCTGTAACACTTATTTTCAACACAGATCATTTTCCTTGTTTCATATCATTAGATATTCCCATTGATTTAAGTAAACACTCAAAGANNNNNNNNNNNNNNNNNNNNNNNNNNNNNNNNNNNNNNNNNNNNNNNAAGGCTGAAATAGATCAAATATTGAATGAAACGCCAAACACCCTAA
GCR_cBin40_Ctg1_186	N	TCACCCACCCTGTACAGTTTCCAGTGGCCTAGTTATAAACAGTTCTCATTTCTCCCTGTACTGCCTGTTGATCCACTAACACGTTTCACTTAATCACATTTCATTAAACACAGACACTACGAGTTGCCTCTGAAGATGACAGACGTGATCAT[A/T]CGCTACACAGAGCCAGATTTGTGCTCCCGACTCTACCACTGACACATCGAGAGCAGTGTTGAAAGCCAGTTTTGCCAGGCACCGTGCAGGACTTTGTCAGGTCTCCTAACAGAGAGAGGGCAACATGGCTGGTTTAAGCAGCTCCATATGGTTCCAGATCTGTGTGCTGTCTCACCAACTCCTGTGGTCATTGATGTGACAATGCTCCATGCAGGATGGATACA
GCR_cBin10148_Ctg1_91	N	TCACCCTGCCCATGCGTATCTTCAGCTTGCTGGGTGTTGAGACAGTAATCCTGACCAA[C/T]GCTGCTGGCGGTCTCAACCAGGACTTCAAGGTGGGAGACATCATGATCATGAAAGACCACATCAACATGCCTGGGTTCGCTGGCAACAACCCACTGTCTGGACCTAACGACGACAGGTAGGCCAGGCAAGCTTGTGTGTTGTGTGTGACAGAGGGAATTACGTTGAACTTGTT
ESTNV_34639_1192	N	TCATGGTTACAGAGCTGCTCAAACTGGCCTAGAAAGTCTGTGGAATCAGAATGCTGATTTGTAGTTNNNNNNNNNNNNNNNNNNNNNNNNNNNNNNNNNNNNNNNNNNNNNNNNNNNNNNNNNNNNNNNNNNNNNNNNNNNNNNNNNNNNNNNCTCTCGCTTAGAATTATGTTTGTTGTTTTCCTGGAGACCATCCTGATCTGATTTTACTACAGTGTCACTTACTGGATTGT[G/T]TAAAAAGCTGAAATAGACATCAGAAGCTACATGTTTCTGTTGCGCCATATGTACTGTGATCTGTGCTACTATCAAAATTGTAATAAAAGTGATGGCAAACAAANAAAAAAAAAAAA
GCR_cBin10025_Ctg1_195	N	TCATTCAACTAGACTCTTTGACAGCATTATCTTCCTTTACAAAATGATGGTACATTTCCTGGTTTACTGCTTGGATCTGAATGTAAGTGTAGACAGCGCTAGTGTAAACCACCGGTAGAATGGAAGCTTGTAAACATTCTACCTAAGTGAAGAGTAGCATGTTTTCTTTGACC[A/C]TAAACAAAGAGTGGACAGTCAACACCGCCATACCCGCAGATAGATATCGTCAGAGGAAGCCATGGGAAGAGCTGGCCATGGTCTGGTCTAG
GCR_cBin24261_Ctg1_69	N	TGACTCAGATGTTTTTCTGGATGCCTGGCTAATAGA[G/T]TTAGAGCCAAACCTTGATAAAAAACAAATACACATATTTTCAGTTTCTCCTGAATCATACATTGAGGTCAAATGAGAATTTTGTGCAAATGTTTGCTTGTATGACAGTTCAACCATTATGGATTCAACCATTATTGATTTTCTGTCATTGTTGGTGTATAGACCATAAGAGGCAACCAATGGAAAGACCATTTCTGTACTTACAAATT
ESTV_14711_157	N	TGGATACTTGACATTCTTTATTTGGCACTAGAAGTGGTTTTACCACTGCTCAAGTATGCCCAAAGATCTTTTGGGGGAATAATTGAAGTACCCTTGAAGGCTCTTTGAAACCCAACATAAAAATTTAAAAAAAGGGAAACTATTTAAACCCAAGTT[C/G]AACACAATGGATACCAGACCGTGACAGTCCACCAATACAGTCTAAGCTTTATCAGTATCCCCTGTTCAAATATAATGGGCCCTTCACACGCAAACGGCGATGGAGCATCATTTTCACACGTAGTTCTATGTACTTTCGTGTGGCTTAAATGTTGGAACGGCCTGTATATGACGCTCCATCGCGCTGTTTGTGTAGCGGCTTTAATTCAGGTCCTCTCTATACCGACGCATACATTTTTTCTTTTTCAATT
ESTNV_32346_630	N	TGTTCTGTTTTTCTATTTCCTGTTTTTTGTTGCTTCTGGTGACTTTGTAAGAGCTGTTTGCAAAGAGGGTCTGTGTTCAGGTACTTTGGATGAAGGGGCAGTGGACGGAGGGGAGTAAGTTAAGTTTCCCCATTATCTCTTTTCCATTCTGTTTCCCAATGGCTTTGTTCAGTTTCTGCCATTCCGGCTCGAAGTTGGGCCAAGATGGCCCACCGCATTCTCATTTAAAGGGGAAATCA[G/T]CAGTTGCTACCTCCATTTTTGGATACATAAATTGATATATACCCATTGATTCTATAAGAATATAACTTATAAATAAATGCTGCGTTAGCTAAGTTCAACAATCGTACCCCATCAGAACCCAAAATATAAAACTTAACATTGTAAACACTCTATAGCCTTAACATGATTAGAACTCATTTTGATATAATGGATGGTCAGTCCTTGCATCCATTTCTGTCTT
BASS123_B7_B04_360	N	TTCATGTTAAAAGTGTGATTTTGGACATTGCATGCACAGACACCATTTTAGGTCACATCGCATAGTTTACATTTGAATTCGTTCCACATGTTTCTCTTTGCTTCCAGGTGCACTACATGGGCATTCGAACACTTCAACACAATCGATTTGCATTTGTACCAGGGAAATCAAAACGAGAACAGGAACAGGTCACCTTCTACATTGTTTGTGTTCACTTGAACATTTCATGTTGGTACCTG[A/G]CAGTCTGTTCTTTATGTAACCACTCCCTTTTCACAGCTCACTAAGGCTTAGACAATGGATGTTGTTTGTTGATGTTTCATGAACGGAACACATTATGTAAACTCTGCCTACTTTCGTCACCAGATATTCTTCATGAAATGCAAGTCGAACATTGTCACAGCAATACATTTAGTTCCAGTGGTTAAACGTGTACTGTAGTTCACGGTTGAGTTGACAGTGTAGCAGGCCATTATGACAACG
ESTNV_34419_496	N	TTTATAATGCACATTGATCAAGGAAACCGCTTATTAAAAAAATCCCATATGGTCCCTTTCCACTAGGTTCTCACTCGTGGACTATTCCTCATATCTAACTTGGACACTATATTCATCCTGTTCTGGGTCAGTTTCCCAAAAGGATGTTATGTTTCTAAGGTGAACCCGGGCCCAGCTCTGTTTGGGGGAACTCATCTGGCTTTCTAACATTGACAACTACTGAGATGTTATATAG[C/G]GCCAACCACTTCTTACTGCATGTTATCGTCAAGCTATTTTCAATCATTTCATTCACAGTTAAATGTCTTCCTGATCTTGTCTGCTAGCTAACAATTTCAACCTCGAACCCCAGAGAAATGGTTATATAGCCAATATTATTCCTTTTCATTCTTCCAAATTAAAAAGTCCCCTCCCATTGTCGTATATGGTCCTTTCTATCTTACCCCAGCGTGTCGACCCCCAGAGTTCCATGCTGCCTA
GCR_cBin1955_Ctg1_60	N	TTTTCTATTTTTCTGCCTGTGGTTTCTT[A/G]TAGGGATATTTACTGGATGTTACGCTGATGAAGTGGCTTCAATAAAAATAAAAAACTTTATTGACATCACAGTAAAATGCTTTCATTTCAACCTTATATCACCTCTAAACATATAGGCTATATGGAAAAGTGTCATTGTACCAAG
ESTNV_31163_234	N	TTTTTTTTTTTGCAAGTTTAAACTTTTATTAATGATATAATAATACAATGATATTTTGTGCAACAGTTTGGTGTGGCGTGGACCTGTACATTATCAACAACAAAACTGCATAAACTCAACACCATGGCAACCTGCAAGCAGAACATCACATGACTTGTACTAGCCTGGGTCCCAGTCTGTTTGTACTCTATTGCCAACTCACTCACTGTCAGTGTCACAGAGCATAAACCGAC[G/T]GGCACTCAGGCTACACCTGTATAGTTTGGCCAAGGTTGTGTCAAAGTGAAAACCGATAGATGTCATCAAGATATACCTACCATGTCATTAAATCCATATACACATAGTTTCCACTAGTATACATTTAAATGGTTGTACTGTATAAGACTACATATGTATATAAAGTGCAAAAAGAAATTAAAACACTTACAGTGAACGCTTGGCCCTGAAGTGCTGTTCGGATGAGTGTGCAAAACCTCC
ESTNV_32170_318	N	AAAGAAATGGGAAAAATGCACAAAAATAAATCAGACAGTTGATGAGAAGGGTGGGTATGGCTGGGGCTTTGGGGTATTTTGGAGTCAGTTCATTAGGACCTCCATCTGCACCAGCCGGGCATTGGTGTCGCCTGCCATGCGGTATGGGATAATCCCAGCAAACGTGATCAGTGCTCGAGCCTGGCTGCGCAGTTGCGAGTCTCTGTCCTCCATGGATCTCTGTGGTCGACCAGGGGTTCCTGCTGTGGC[C/G]CCCTTTAGACGTTTGTACTGCATGAACAGGATGTTCATGGTGGCCAGGAGAACCTCCGACAAATTATGCCTGACCTCATCACTGAAATTACGGAATGCAGCGACCCTCTCCTCCACACTGTCCTGACTCAGAGGGACCAGCTTCAGTCTCTCCATAACATCGTAGGCCTGGTCTATGTGACCAACATGGTACTCATCAAAGAAGGTCATGAGGTCCAGCAGCAGGTAGAAGGTGCTGTCCACAGACTTCT
GCR_cBin21396_Ctg1_111	N	AACAAACTGGAATTCAAGTAATTGAACTGAAGTCAGTCATTTAGTTGTTTAATAATAATAATAATAGACATGTTGGTTCATCGCTCTAACACTAACACACACACAGAAAT[C/G]GACATGAGATGTTCAGTTGTGTGTTCTGCCCTAATGGTTCTAAAAGAAGGATAATAAAACCTTGTGTTCTAAAAACCATGGAACGAACGTGACAAGAAAGAAAAAAGGGCGAAAATGGAAATGAATCAATTTCCCTATGATCCCTTAGAATAAAGAACAAATAAAATAAACCTTCAAACAAAATGTATGAATGACAACAGGATATCATTTCCATTTCCATTCTCACATCAGACCTAGCGA

ESTNV_29115_481	N	AAGACTATAGCTAGATAATAATGCATTGGACATCTCAGTAAGTGCTCAACCCAGAGTTGAACCTCTTAGATTCTCTTTTTGTTCTCAGTCCTTTCAGCTAAGTAAGTGCTTTACATTACTAGCAATGCAACTTTTAT CTGATATTAGATCAATGATTGGACCATGTGATTGAAGTCCACCCTACTGATTTGCCATAAGGTAAAGAGACACGTGCAAACTGCATCTAGGTTTTGCAGTTCT[A/C]TAGATATGTTGAAACAATTGATGTCCAA AGGTTCTTCTGCATAGGTCACTGGATTAGGGGGGCAATGAATGTACAAATTCCCTAATAATGTTGGGCAGGTGAGGGTCTTTGTACAATCCATTGTGAAGGTACCCAAGGGATCTGGCGAACGGTACCATCTT AACTCGATTCTCTGAGTTTTGGGCGCTCTGTGCAATTTCATACAGCTGCTGAGCCATGTGGATAGGCACTATGTGGTCA
ESTNV_31226_313	N	AATTTGTATACGCCTAGTAAAACACAATAGTTACTGCGCTAATAATGTTGGGTCCAGCTTCCTGAGGCACCAGGACATTGTGTTGGGGCTGCTCTATGGAACCCGTCCTACTATTCTATGGGGAAGAAAGAAAT GTACAACTGTCTGTTTCTCTCATTGACCTGTCTATGCATTCTCCCGGGTTATGATTCAGAGGAATGTGTGACTTCTGTCTTTGCAAAGAGAAGTTCTGTTGACGTACC[A/C]AAGGGGGGCACTCTTTCCTTATCC TGTGTTGTTCAGCATTGTGGAGATGATGGCTGGACAGGGGGATGGGGGCTGTCAACAGAGGGACAGTTCCTTCTCTTTAGTCCCACTCCAAGGCATCATCTCTCCAAAGTCACACTGACAACCAATAGTACTCG TCTGCTCATGNACATCCTCAACGTCAACCAATCAGATCACGGACTGTACCAATGCCAGATCACCTGGGTTGAGGGATCCACC
ESTNV_18603_371	N	AAAAGAGGGTTAAAGAAAATGAGCATTTCTTGTCAGTTCCGTTTTGGTGCCAAATGAATATGACCCAGTCTTGACTGAATAGGCCGACTGCCAGTACCCAGTTTGTCCACGATGTTTCTGTTTTTGGTAGCGATT TGATACGACCTGTGGTTCAATTAAGACCTGATAAACTTCCTCCTTCCTTGCTGATTCTACTTTTACCCCTCTATTTTTATTTTTACCCCTCTCTTCTTCTTCTTC[A/T]GTAGAAGTTACCACGATAACAGGGTTTTCGT AGCCCAACCTTCTCCTTTTTTGGGGGGGGGGG
ssa25_28664700-28665100_VGLL3_AKAP11_intergenic_TOP_VIP	F	AAGTCTTATCACTGTGATGTTCTATGGGACATGTTCAATCAGAATCTGCAACCAGCGGTAGAGTTTGACTGAATGTGAGTCCTGCTCAAGGAGCTCCTACCTCTGGTTCCACTGGCGTGCTACTATGGCTACCTG GTCCCTGAGACATACACCAATAAGATAAACAATAGTTTCTCCTCTGTTGTCATCCAGAATTAATC[A/C]GATTGTATTCCTCCAGTACAGAACAGCTGTGTGGTCTGATTGGGTTAATGACAGTGTGATGTGGGA ATGTCTGTTAAACATAGAACACTCTGCATTCACACAGCCAACAATAACCCCACTACTAAACACTGTGACATTTATTGATGGGATTTTAACAGCCTGTTAAATGGTTGTGGTAGATGGACAGCCAGAAATGGAGG
ssa09_24870037-24870437_PPM1a_SIX6_intergenic_TOP_VIP.fasta	F	AACAGGCTGAATTTCCCTTTGTCCCCACTCTCCTCCATAGCAAGGATGCTACCAGGGAATATCGAGGCTCTATCTGCTCCTTATCATACAAATACAGCATCAATATCAAAGGGAACGAACGGTAAGATACATTGG ACCCGTCTCAGAATTTCAAAGTATATTATATTACAAATAATATAATACAGTGTGGTGCTCTGTAT[A/G]GTTAGTTTTATTTTCCACTATAATATTTAACTTCTGGTAAGATCTATGAATTATAGACAGTCATTTTTG TTACCAAGGACATTTCATTGAATTAATCTGCACAGCAGTGACATTATGCCTTGTATAAAGTATACCTTTACTACCTATTCCTACTAACAGTATTAAATATATGCACAAACTGGCGCAGAAGGAGATGTCGT

*N=neutral, F=functional

Datasets and code underlying the study

Eira_Genotypes.csv Includes phenotypic data and genotype matrix for 346 individuals caught in River Eira.

Stryn_Genotypes.csv Includes phenotypic data and genotype matrix for 214 individuals caught in River Stryn.

Discharge.txt Waterflow in River Eira presented as daily waterflow (m^3/sec) in River Eira during 1931-2016.

Atlantic salmon of the river Eira 1925-2016.csv Metadata for 8324 adult Atlantic salmon caught by anglers in River Eira during the periods 1925-1926 and 1940-2016 (except 1958-1965).

Atlantic salmon of the river Stryn 1942-1993.csv Metadata for 226 and 2849 adult Atlantic salmon caught by anglers in River Stryn during the periods 1942-1957 and 1970-1993, respectively.

Carlin_Tagged_Eira_Genotypes.csv Includes phenotypic data and genotype matrix for 79 Carlin tagged Atlantic salmon recaptured at sea as sexually mature adults during 1961-1967.

<u>Code:</u>
Drift_WF_approximation_Zenodo.R: Code to determine whether temporal changes in *vgll3* and *six6* allele frequency in the Eira and Stryn populations could be explained by genetic drift.

eira_adaptdyn.R and **eira_adaptdyn.cpp:** Computer code for the adaptive dynamics model.
